# Mapping the Evolutionary Space of SARS-CoV-2 Variants to Anticipate Emergence of Subvariants Resistant to COVID-19 Therapeutics

**DOI:** 10.1371/journal.pcbi.1012215

**Published:** 2024-06-10

**Authors:** Roberth Anthony Rojas Chávez, Mohammad Fili, Changze Han, Syed A. Rahman, Isaiah G. L. Bicar, Sullivan Gregory, Annika Helverson, Guiping Hu, Benjamin W. Darbro, Jishnu Das, Grant D. Brown, Hillel Haim

**Affiliations:** 1 Department of Microbiology and Immunology, The University of Iowa, Iowa City, Iowa, United States of America; 2 Department of Industrial and Manufacturing Systems Engineering, Iowa State University, Ames, Iowa, United States of America; 3 Center for Systems Immunology, Departments of Immunology and Computational & Systems Biology, University of Pittsburgh School of Medicine, Pittsburgh, Pennsylvania, United States of America; 4 Department of Biostatistics, College of Public Health, The University of Iowa, Iowa City, Iowa, United States of America; 5 Department of Pediatrics, University of Iowa Hospitals and Clinics, Iowa City, Iowa, United States of America; University of Connecticut School of Medicine, UNITED STATES

## Abstract

New sublineages of SARS-CoV-2 variants-of-concern (VOCs) continuously emerge with mutations in the spike glycoprotein. In most cases, the sublineage-defining mutations vary between the VOCs. It is unclear whether these differences reflect lineage-specific likelihoods for mutations at each spike position or the stochastic nature of their appearance. Here we show that SARS-CoV-2 lineages have distinct evolutionary spaces (a probabilistic definition of the sequence states that can be occupied by expanding virus subpopulations). This space can be accurately inferred from the patterns of amino acid variability at the whole-protein level. Robust networks of co-variable sites identify the highest-likelihood mutations in new VOC sublineages and predict remarkably well the emergence of subvariants with resistance mutations to COVID-19 therapeutics. Our studies reveal the contribution of low frequency variant patterns at heterologous sites across the protein to accurate prediction of the changes at each position of interest.

## Author summary

We describe a systematic approach to identify clues that can predict the mutational profiles of SARS-CoV-2 subvariants at early stages after emergence of their parental lineages. We found that the likelihood for mutations at each position of spike is lineage-specific, and is estimated well by the mutational patterns at all positions of the protein. As examples, we apply the model to forecast emergence of resistance to the antibody therapeutics Bebtelovimab and Cilgavimab, which appeared in sublineages of VOCs BA.5 and BA.4, respectively. Emergence of these subvariants required changes in treatment protocols of COVID-19 patients. In both cases, the escape mutations were predicted well by the mutational profiles at all positions of spike at early time points after emergence of the parental VOCs. Accurate estimations of the imminent changes in SARS-CoV-2 lineages can contribute to design and selection of therapeutics that maintain their efficacy against future forms of this virus.

## Introduction

Since emerging in December 2019, SARS-CoV-2 has caused devastating effects worldwide. By March 2024, more than seven million deaths have been attributed to the infection [1] and estimated economic losses of more than $10 trillion [2]. Several SARS-CoV-2 VOCs have appeared during the pandemic, all containing mutations in the spike glycoprotein that adorns the virus surface [3–5]. Spike mediates fusion with host cells and is the primary target for antibodies elicited by infection or vaccination [6]. Changes in spike can increase virus infectivity, transmissibility, or resistance to host antibodies and therapeutics [7]. New sublineages of the VOCs continuously appear with such mutations [8–10]. In some cases, the sublineage-defining mutations are convergent, whereby the same substitution occurs independently in different expanding virus populations [11–13]. Such changes are usually guided by positive selection pressures [14–16]. By contrast, many mutations that define the VOC sublineages are found at different positions of spike in the diverse VOCs. This observation raises two questions. First, do the distinct patterns of mutations that emerge within different lineages reflect the stochastic nature of their appearance or lineage-specific likelihoods for their emergence? Second, if not driven solely by stochasticity, can we identify clues that will inform of the evolutionary space of each lineage? Answers to these questions are critical for development and selection of therapeutics that are effective against virus forms expected to emerge in the future.

To address the above, we analyzed spike sequences from diverse SARS-CoV-2 lineages collected since the beginning of the COVID-19 pandemic. Clear lineage-specific mutational spaces were observed, defined by the patterns of amino acid variability at spike positions among strains closest to each VOC ancestor. We then compared the mutational space of spike in each VOC with its evolutionary path (i.e., the collection of mutations that define its descendant sublineages). The sites of change in the emergent sublineages were characterized by high variability in the parental lineage and a high variability “environment”, composed of adjacent positions on the protein structure and at neighboring nodes on the network of co-variable sites. Combined, these measures of positional and environmental variability predicted well the mutational profiles of the new sublineages that emerged from each VOC. Similarly, the mutations in subvariants of VOCs BA.4 and BA.5, which rendered the virus resistant to antibody therapeutics and resulted in changes in the treatments recommended for COVID-19 patients, were predicted remarkably well by the first collected sequences of the parental VOCs. These studies reveal the diversifying nature of the evolutionary space of spike and the predictability of the changes that give rise to new SARS-CoV-2 spike forms.

## Results

### The mutational space of SARS-CoV-2 spike is lineage-specific and diversifying

**Fig 1:**
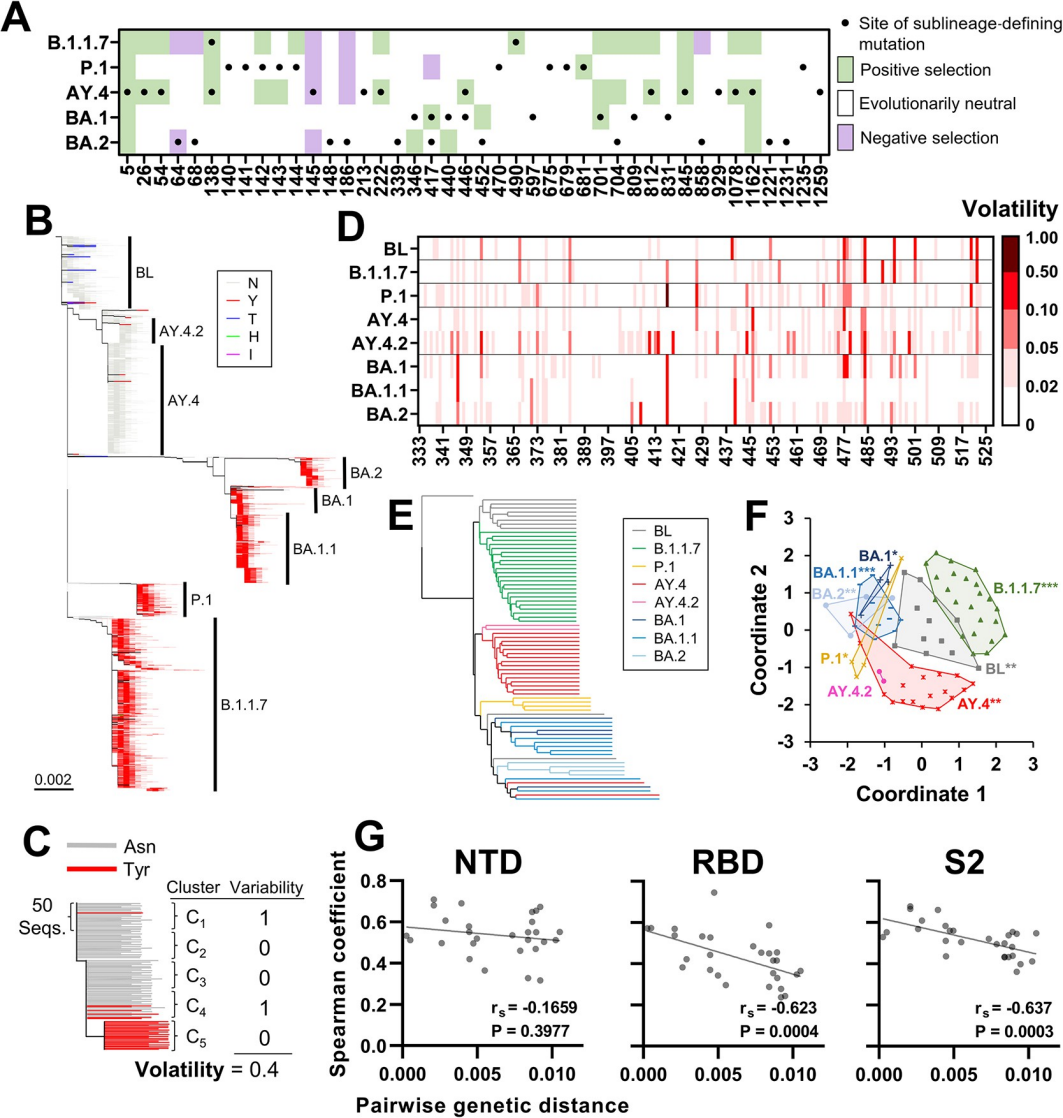
The mutational space of spike is specific for SARS-CoV-2 lineage. **(A)** Black dots indicate sites of mutation that define VOC sublineages and emerged until April 8^th^ 2022. Cells are shaded by the inferred selective pressures applied on the sites in the baseline of each VOC calculated using the SLAC method. **(B)** Phylogenetic tree based on 40,350 unique spike sequences from the indicated lineages. The SARS-CoV-2 baseline (BL) group is composed of sequences within 0.0015 nucleotide substitutions per site from the SARS-CoV-2 ancestral strain. Branches are colored by the residue at position 501. **(C)** Schematic of the approach to calculate volatility for each position of spike. **(D)** Volatility at RBD positions in the indicated VOCs or the BL group. **(E)** Lineages were partitioned into groups of 500 sequences and the absence or presence of volatility at all spike positions in each group was determined. All groups were thus assigned 1273-bit strings that describes the volatility profile of spike. Strings were compared using the UPGMA clustering method. **(F)** Relationships between the 1273-bit strings. Data points represent the strings of all 500-sequence clusters, which are labeled by lineage. To visualize these relationships, the distance matrix between all vectors was used as input for multidimensional scaling. Lineage-specificity of the profiles was determined by a permutation test. P-values: *, P<0.05; **, P<0.005; ***, P<0.0005. **(G)** Volatility was calculated for each lineage at all positions of the NTD (20–286), RBD (333–527) and S2 (686–1213). The Spearman correlation coefficient between volatility values in any two lineages was determined. Coefficients are compared with the mean nucleotide distance that separates any two lineages. r_S_, Spearman coefficient. P-values, two-tailed test.

We examined the evolutionary path of spike in different VOCs, defined by the mutations that dominate their emergent sublineages. Using lineage classifications of the Pango system [17], we compared the sublineage-defining mutations that emerged until April 8^th^ 2022 in VOCs B.1.1.7 (Alpha), P.1 (Gamma), AY.4 (Delta), and BA.1 and BA.2 (Omicron) (**Fig 1A**). Limited similarity was observed between the sublineage mutation profiles within the different VOCs–only three of the 42 mutations appeared in more than one VOC. We also calculated for these positions the synonymous and nonsynonymous mutation rates to infer the sites under selection in each VOC. To this end, we analyzed sequences that are phylogenetically closest to each lineage ancestor (see sample collection times for all lineages in **S1A Fig**). All Pango-designated sublineages of the VOCs were removed from the datasets. In addition, to avoid contamination of the parental lineages by misclassified sequences from emergent sublineages, all datasets were manually inspected and curated (see Methods Section). Spike sequences that appeared at least twice in the population and lack any nucleotide ambiguities were aligned and “compressed” to obtain a single representative for each unique sequence. To infer selection pressures, we used the Single-Likelihood Ancestor Counting (SLAC) method [18]. This approach, which assumes that selection pressures applied on the positions are constant, was used given the relatively low level of divergence within each VOC, the limited time range of sample collection for each VOC, and the large size of the datasets. Many sublineage-defining mutation sites did not show evidence for positive selection in their parental lineages using the SLAC method (**Fig 1A)** or other approaches that are based on the dN/dS metric (**S1B Fig**). Thus, while there is strong evidence for a selective advantage at many of these sites, such as appearance of convergent mutations in different phylogenetic contexts and geographic locations [11,19], as well as documented effects on resistance to vaccine-elicited antibodies [20–22], inferences of selection pressures alone may not be sufficient to identify all mutations that emerge in the VOC sublineages.

To better understand the distinct evolutionary paths of the VOCs, we also examined the mutational spaces of spike in the above parental lineages, defined as the collection of sites that exhibit variability in amino acid sequence. As a group that represents isolates closest to the SARS-CoV-2 ancestral strain (designated the SARS-CoV-2 “Baseline”), we used sequences within 0.0015 nucleotide substitutions per site from the SARS-CoV-2 spike ancestral sequence (accession number NC_045512) [23]. Lineages in the SARS-CoV-2 Baseline group contained four or less spike mutations relative to the SARS-CoV-2 ancestor and none of them were VOCs. In addition, as controls, we included sublineages BA.1.1 and AY.4.2, which emerged from lineages BA.1 and AY.4, respectively. Evolutionary relationships among the sequences were inferred, and a maximum likelihood phylogenetic tree was constructed (see Methods section and **Fig 1B**). To quantify amino acid variability at spike positions in the above groups, they were partitioned into clusters of 50 sequences (see Methods Section, and optimization of cluster size in **S2A** and **S2B Fig**). The proportion of clusters that contain any variability in amino acid sequence at each position was designated “Volatility” (see schematic in **Fig 1C**). This simple variable was designed to quantify the frequency of independent substitution events at each site.

Considerable differences were observed between the volatility profiles of spike in the diverse lineages (see RBD positions in **Fig 1D** and all positions in **S2C Fig**). To compare the profiles, we partitioned each lineage into groups of 10 clusters (500 sequences). The absence or presence of amino acid variability at each spike position was determined among all group sequences, and the groups were assigned 1273-bit vectors that describe their volatility profiles for the entire spike protein (see schematic in **S2D Fig**). Jaccard distances between the vectors were used to determine hierarchical relationships by the unweighted pair group method with arithmetic mean (UPGMA) approach [24,25]. Clear clustering of profiles from the same group was observed (**Fig 1E**). To determine the statistical significance of these patterns, Euclidean distances were measured between all vectors, and intra-lineage distances were compared with inter-lineage distances using a permutation test [26]. As shown in **Fig 1F**, all groups (except the smaller AY.4.2) exhibited significant specificity of their volatility profiles.

We then asked whether phylogenetically closer lineages exhibit similar volatility profiles. Positions of the RBD, N-terminal domain (NTD) and S2 subunit of spike were analyzed separately. For the RBD and S2, negative relationships were observed between the genetic distance that separates any two lineage founders and the correlation between their volatility profiles (**Fig 1G**). For the NTD, which contains a relatively high proportion of sites with mutations, such a relationship was not observed. These findings suggest that the mutational space of spike is specific for each lineage and is diversifying. As such, and assuming that the mutational space is related to the evolutionary path of the virus in the population, these findings also suggest that each lineage may have a distinct set of changes that can appear in descendent sublineages. This notion was supported by the finding that the sites of sublineage-defining mutations often showed higher volatility in the baseline of the lineage from which they emerged relative to other lineages (**S3A Fig**).

### A high volatility state increases the likelihood of each spike position to emerge with founder mutations in new lineages

Several sites of sublineage mutations exhibited strong evidence for positive selection in the parental VOC, often associated with high volatility values (**Figs 1A** and **S3B**). Nevertheless, some were inferred to be evolutionarily neutral (or even under negative selection) and exhibited low volatility in the VOC baseline from which they emerged. Thus, the frequency of independent mutations within each lineage (volatility) or inferences of selective pressures show only modest ability to predict the sites of mutation in sublineages that emerge from each VOC. We sought to identify clues that would accurately forecast such events. To this end, we used a systematic approach, divided into the following steps: **(i)** Identify variables that contribute to predicting the mutation sites that define the VOCs using sequences closest to the SARS-CoV-2 ancestor, **(ii)** Develop and test a model that combines these variables, **(iii)** Apply the model to sequences from the VOC baselines to forecast VOC sublineages, and **(iv)** Validate the model using independent datasets to forecast resistance to COVID-19 therapeutics in sublineages of recent VOCs.

**Fig 2:**
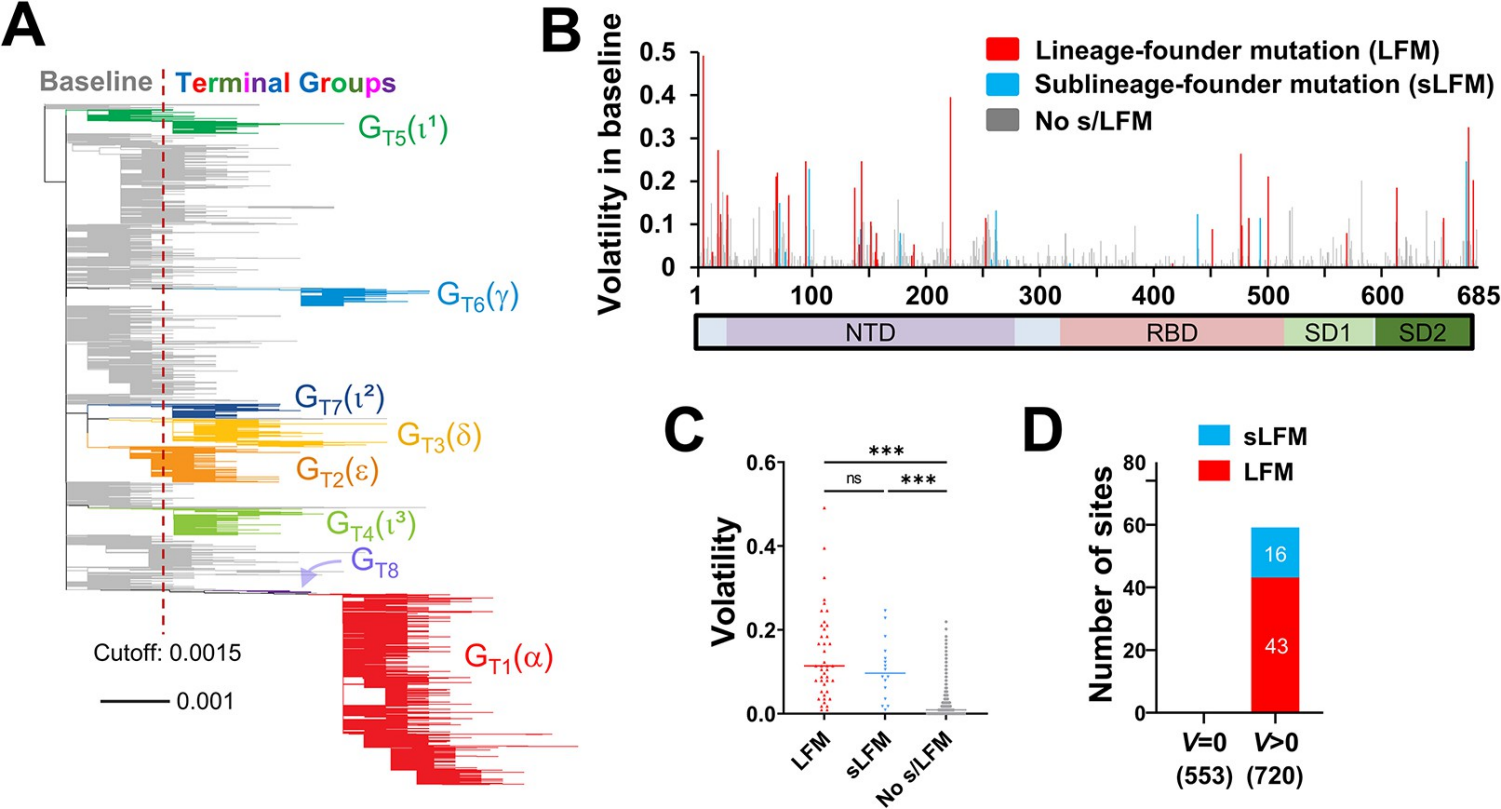
Spike positions with high volatility in the baseline group emerge as sites of sub/lineage-founder mutations. **(A)** Phylogenetic tree based on 16,808 unique spike sequences. Terminal groups are colored and labeled, with their WHO variant designations in parentheses. **(B)** Volatility values for all positions of spike subunit S1 calculated using the 114 baseline clusters (see values for subunit S2 in **S4B Fig**). **(C)** Comparison of volatility values between spike positions that emerged with LFMs, sLFMs or no such mutations. P-values in an unpaired T test: ***, P<0.0005; ****, P<0.00005; ns, not significant. **(D)** Number of positions that appeared with LFMs and sLFMs when volatility in the BL group was zero or larger than zero. The number of positions in each subset is indicated in parentheses.

First, we examined if sites with high volatility are more likely to emerge with lineage-defining mutations relative to sites with low volatility. For this purpose, instead of Pango lineage designations (determined by all virus genes), we classified the sequences based only on the spike gene. Nucleotide sequences of 615,374 isolates from samples collected worldwide during the early pandemic (December 2019 to July 2021) were used. Only sequences that appear at least twice in the population and lack nucleotide ambiguities were included. Evolutionary relationships among the sequences were inferred, and a maximum likelihood tree was constructed (**Fig 2A**). We then partitioned the tree into discrete groups separated by a minimal distance of 0.004 substitutions per site. As expected, many groups corresponded to known VOCs (**S1 Table**). The Baseline groups (closest to the SARS-CoV-2 ancestor, G_B1_-G_B12_) were distinguished from the Terminal emergent groups (G_T1_-G_T8_) using a threshold of 0.0015 substitutions per site between the centroid of each group and the spike ancestral sequence.

Volatility of each spike position in the Baseline was compared with the absence or presence of two types of mutations at the site in any of the Terminal groups: **(i)** LFMs (Lineage Founder Mutations), which are substitutions in the group ancestors (relative to the SARS-CoV-2 ancestral sequence) and in at least 50% of all sequences from that group, and **(ii)** sLFMs (sub-Lineage Founder Mutations), which are not found in the group ancestor and represent clonal expansions that dominate at least one 50-sequence cluster (see examples in **S4A Fig**). A total of 43 LFMs and 16 sLFMs were detected in the Terminal groups (see **S1 Table**). With this distinction, we sought to capture changes analogous to the mutations that define the VOCs and the VOC sublineages (as classified by the Pango system). Each spike position was counted as an LFM or sLFM only once, whether it appeared in multiple groups due to convergence (such as the mutation at position 501) or due to common descent (such as positions 5, 95 and 253 in the three Iota groups, see **S1 Table**). Any position that appeared as an LFM was not counted as an sLFM. We observed that most positions with high volatility in the Baseline emerged with LFMs or sLFMs (s/LFMs, see positions of subunit S1 in **Fig 2B** and of subunit S2 in **S4B Fig**). Among positions with the highest volatility values, most appeared as s/LFMs in at least one group (**S4C Fig**). Sites of s/LFMs were more volatile than sites with no such mutations (**Fig 2C**). Furthermore, non-volatile sites in the Baseline did not emerge with s/LFMs in any Baseline or Terminal group (**Fig 2D**). Therefore, a high level of volatility in the Baseline group precedes (as inferred phylogenetically) the emergence of s/LFMs in the descendent lineages (see performance in **S4D Fig**). These findings were not surprising–a higher frequency of independent mutation events at any spike position is expected to increase the likelihood of the site to contain a founder mutation in any newly-emerging lineage [27,28].

### A high volatility environment increases the likelihood of spike positions to emerge with sub/lineage founder mutations

**Fig 3:**
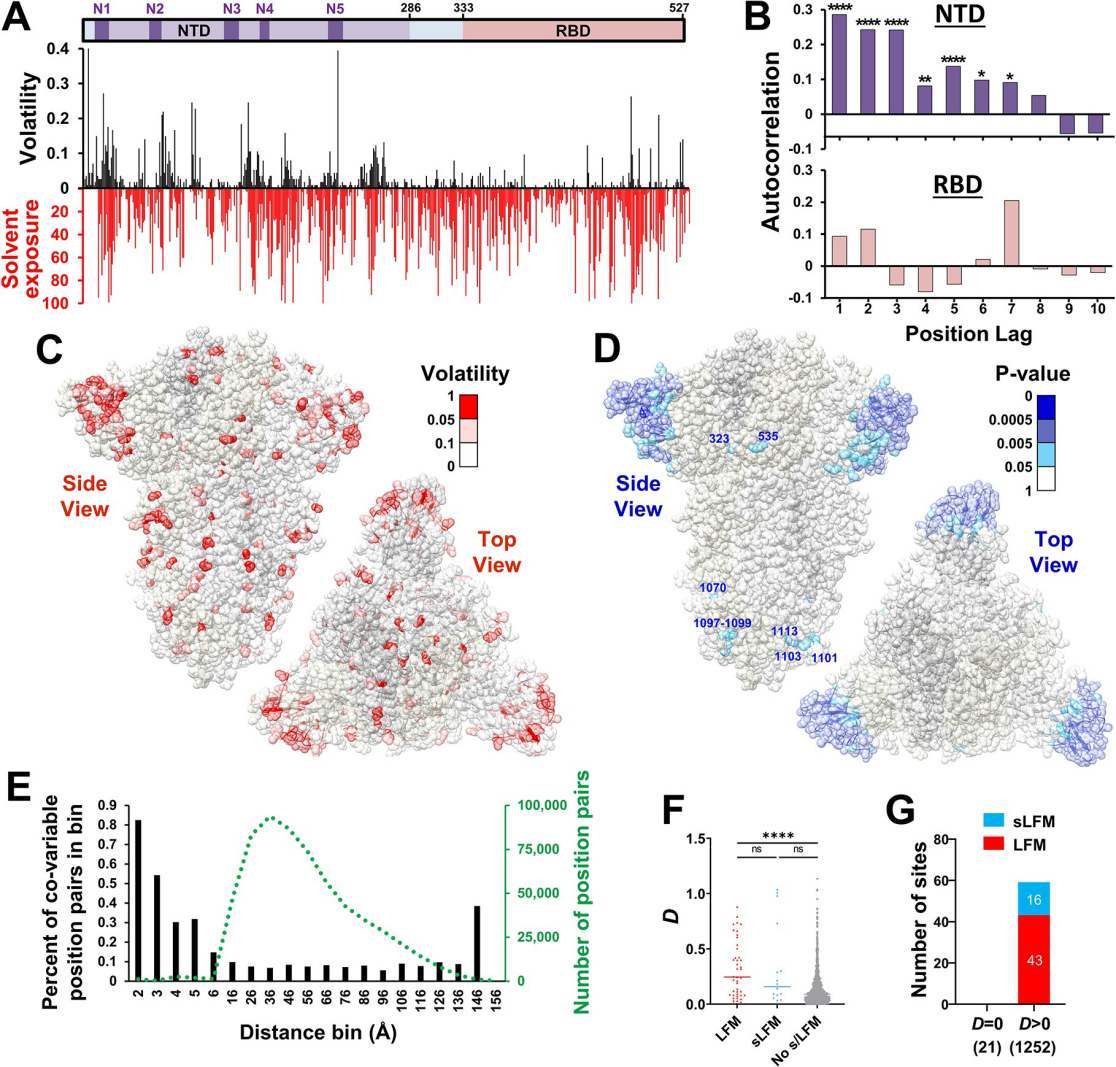
Spike positions in a volatile environment emerge as sites of sub/lineage-founder mutations. **(A)** Volatility values for positions 1–527 were calculated for the SARS-CoV-2 BL group (black bars). Solvent exposure of the residues (based on spike structure 6ZGI) is expressed as a percent of the total solvent-accessible surface area of each residue (red bars). Positions unresolved on the structure (1–13 and 71–75) are assigned exposure values of 0. **(B)** Autocorrelation of volatility values for the NTD and RBD. Significance of the autocorrelation was calculated using the Spearman rank-order test: *, P<0.05; **, P<0.005; ***, P<0.0005; ****, P<0.00005. **(C)** Mapping of volatility values calculated for the BL group onto the spike trimer structure. **(D)** Results of a permutation test to identify positions with high volatility at their 10 closest neighbors on the trimer structure. Low P-values indicate sites with a high-volatility environment. Such sites located outside the NTD are labeled. **(E)** Fisher’s exact test was used to calculate the co-occurrence of a volatile state at any two positions of spike in the 114 clusters of the SARS-CoV-2 BL group. The distance between the closest atoms of any two residues was also calculated. Bars indicate the percent of position pairs with significant co-variability (P-value < 0.01) as a percent of all position pairs separated by the same distance range. The number of position pairs in each bin is indicated by the dotted green line. **(F)** The variable *D* describes for each position the total distance-weighted volatility at spike positions within 6Å on the trimer structure. *D* values are compared between positions with LFMs, sLFMs or no such mutations. **(G)** The number of positions that emerged with LFMs and sLFMs when the *D* value was zero or larger than zero. The number of positions in each subset is indicated in parentheses.

We examined whether, in addition to the volatility level at each site, the likelihood of any spike position *i* to emerge with s/LFMs can also be inferred from the volatility profile at heterologous sites *j*. To this end, we first analyzed the relationships between volatility of sites along the linear sequence of the protein. Several clusters were observed in the NTD, whereas less or none were observed in the RBD (**Fig 3A**). The volatile clusters in the NTD corresponded only partially with the location of the five solvent-accessible NTD loops N1-N5 [29]. Accordingly, the correlation between volatility values and relative solvent exposure of the residues was significant but not linear (**S5A** and **S5B Fig**). To quantify the clustering of volatility on the linear sequence, we tested its autocorrelation using lags of 1 to 10 positions. This test measures the relationship of the variable with position-lagged values of itself, whereby a positive autocorrelation describes “persistence” of the volatile state across positions. Consistent with the patterns observed in **Fig 3A**, a positive autocorrelation was detected for the NTD, with a gradual decrease in significance for increasing lags, and loss of significance at a lag of 8 positions (**Fig 3B**). By contrast, the RBD did not show significant clustering of volatile sites.

Spike proteins on the surface of virus particles are folded and arranged as homotrimers [30]. Accordingly, we also examined the clustering of volatility on the three-dimensional structure of the protein. To this end, volatility values were mapped onto the cryo-electron microscopy structure of the ancestral SARS-CoV-2 spike trimer (**Fig 3C**) [31]. Consistent with the patterns observed for the linear sequence, clustering of volatile sites was primarily detected in the NTD (**Fig 3D**), with a limited number of clusters in the S2 subunit, and none in the RBD. We further analyzed the spatial clustering of volatility by comparing the distance that separates any two positions and the co-occurrence of a volatile state. To this end, we used the 114 clusters of the SARS-CoV-2 Baseline group to determine the covariance of volatility at any two positions. P-values for the association between volatility of all position pairs were calculated using Fisher’s exact test. We then compared the distance that separates the closest atoms of any two residues with the statistical significance of their co-variability. Sites separated by less than 2Å, mainly representing contiguous sites on the linear sequence of the protein (distance ~1.3Å), exhibited a relatively high frequency of co-variable pairs (**Fig 3E**). Sites separated by 2Å to 6Å showed a gradual decline in the frequency of co-variability. In addition, albeit with a low frequency, significant associations were observed between some position pairs separated by distances greater than 6Å.

We hypothesized that if the spatial clustering patterns are “stable” over time, then the volatility in the environment of each position may capture its likelihood for future changes. To test this hypothesis, we generated a variable (designated *D*) that describes for each position *i* the total distance-weighted “environmental” volatility:

$$D_{i}=\sum_{s=1}^{n}\frac{1}{\Delta_{is}}∙V_{s}$$ [1]

where *n* is the number of positions *s* within 6Å of position *i*, Δ_*i*s_ is the distance between the closest two atoms of position *i* and each position *s*, and *V*_s_ is the volatility at each position *s*. Similar to the volatility values, *D* values were higher for positions that emerged with s/LFMs (**Fig 3F**). Furthermore, positions with a non-volatile environment (i.e., *D* = 0) did not emerge with s/LFMs (**Fig 3G**). Performance of the *D* variable to predict s/LFMs was modestly higher than that of the eight adjacent sites on the linear sequence of the protein (**S5C–S5E Fig**). Therefore, high volatility at any position in the SARS-CoV-2 Baseline and high volatility at adjacent positions on spike increase the likelihood of the site to emerge with s/LFMs in descendent lineages.

### High volatility at primary nodes on the network of co-variable sites is associated with emergence of sub/lineage founder mutations

**Fig 4:**
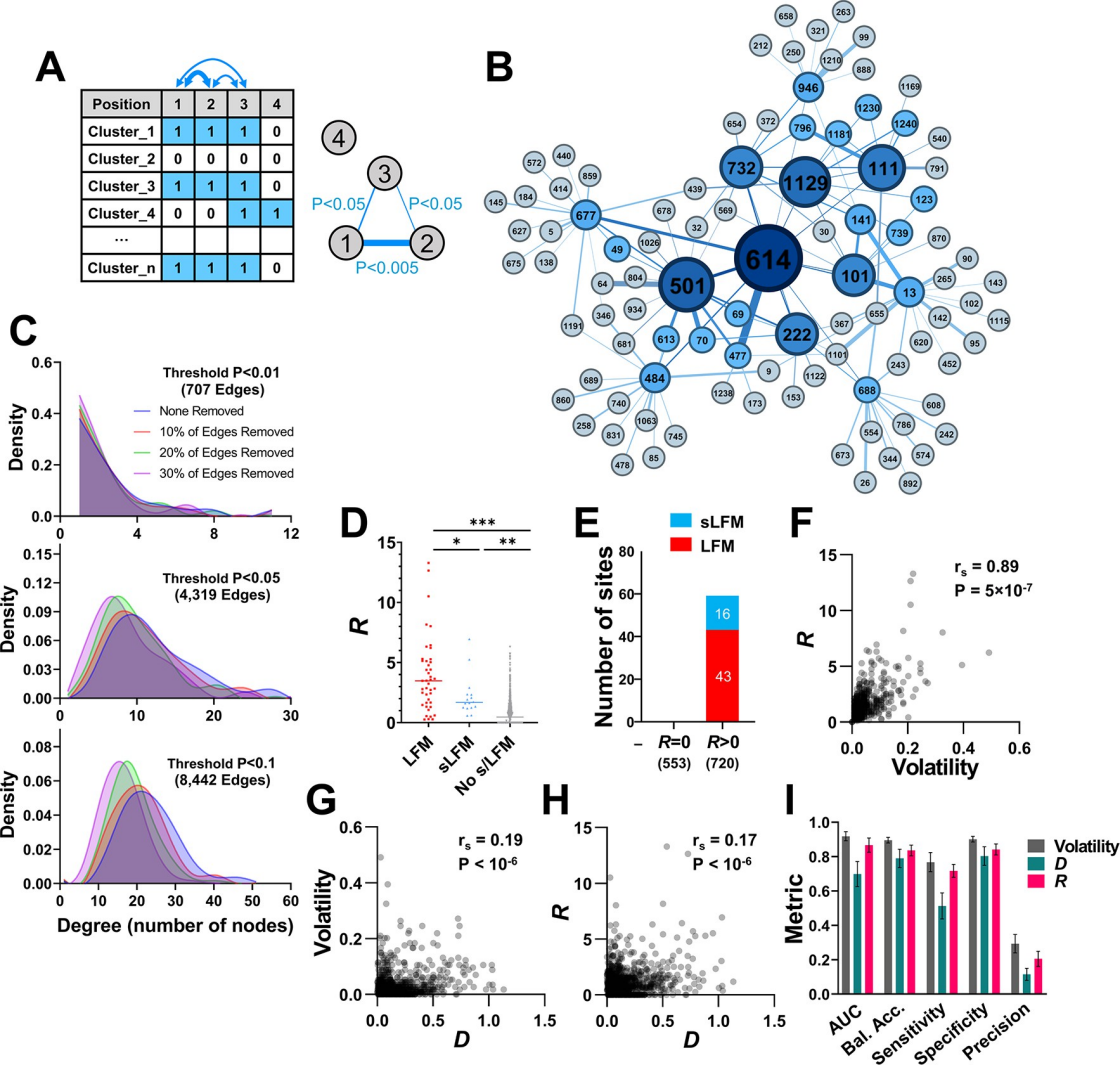
High volatility at co-variable sites is associated with emergence of LFMs and sLFMs. **(A)** Schematic of the approach to generate the co-variability network of spike. For all positions, the absence (0) or presence (1) of amino acid variability was determined in each cluster of 50 sequences. The co-occurrence of variability at all position pairs was calculated using Fisher’s test, and the P-values were used to construct the network. **(B)** The co-variability network around position 614 as the root node. Edges were assigned if P-values were smaller than 0.05. First- and second-degree nodes are shown. Node size corresponds to the number of triangle counts for each position. **(C)** Network robustness. Networks were constructed using P-value thresholds of <0.01, <0.05 or <0.1. For each network, we randomly deleted 10%, 20% or 30% of edges and examined the effect on network stability. The degree distribution (i.e., the number of nodes associated with each position) is shown for the intact and depleted networks. **(D)** *R* values describe for each position the total weighted volatility at network-associated positions. *R* values for spike positions that emerged with LFMs, sLFMs or with no such mutations are shown. **(E)** Number of LFMs and sLFMs that emerged at spike positions when *R* in the BL group was equal to zero or greater than zero. **(F-H)** Correlations between volatility, *D* and *R* values. r_s_, Spearman coefficient. P-value, two-tailed test. **(I)** Classification metrics for evaluating performance of volatility, *D* and *R* values to predict presence of s/LFMs using univariate logistic regression. Error bars, standard errors of the means for five-fold cross-validation. Bal. Acc., Balanced Accuracy.

Given the observed associations between volatility of many non-adjacent sites on the spike trimer (**Fig 3E**), we sought to generate a variable similar to *D* that applies P-values of the co-variability analysis as a measure of the “distance” between spike positions. To this end, P-values calculated by Fisher’s exact test were used to construct the network of co-variability, whereby the edges that connect the nodes (positions) are defined by the statistical significance of the association between their volatility profiles (see schematic in **Fig 4A**, an example of a network segment in **Fig 4B,** and distribution of the P-values in **S6A Fig**). To determine the significance threshold to apply for network construction, we examined structural properties of the network and its robustness to random deletion of edges. Two network topological metrics were assessed: **(i)** Degree distribution, which describes the average number of connections each node has with other nodes, and **(ii)** Closeness centrality, which describes for each node the sum of the path lengths to all other nodes in the network (more central nodes have lower values) [32]. For robust scale-free networks, limited random edge deletions only minimally perturb their topological properties [33]. We found that networks defined at a more stringent significance threshold (P<0.01) contained considerably less information than higher thresholds, reflected by lower degree distribution and closeness centrality values; however, the network was more robust to random edge deletions (**Figs 4C**, **S6B, and S6C**). By contrast, when less stringent significance thresholds were used (P<0.1), the number of edges was greater (i.e., more information was contained regarding the co-variable positions); however, the network was less robust to edge deletions. This suggested that an intermediate significance threshold (P<0.05) would provide a sufficiently stable network without losing most information. To better understand the information contained in this network, we randomized volatility values among clusters (while maintaining the same level of volatility for each position), and then constructed new networks based on the permuted data. As expected of an informative dataset, the original network was more complex and contained a larger number of high-degree nodes than the randomized networks (**S6D Fig**).

We then examined whether, for any spike position *i*, the presence of high volatility at its network-neighboring (co-variable) sites *q* is associated with emergence of s/LFMs. To this end, we generated a simple measure (*R*) designed to capture for each position *i* the total weighted volatility of its network-neighbors *q* (*q*_1_, *q*_2_, *q*_3_ *… q*_n_), using a P-value of 0.05 as the threshold:

$$R_{i}=\sum_{q=1}^{n}w_{iq}∙V_{q}$$ [2]

where *V*_q_ is the volatility at each position *q* calculated using the Baseline sequences, and *w*_iq_ is the evidence for association between volatility of position *i* and each of its positions *q* (calculated as the -log_10_(P-value) in Fisher’s test). Similar to the volatility and *D* values, *R* values were significantly higher for positions with s/LFMs relative to positions with no such mutations (**Fig 4D**). Furthermore, an *R* value of zero in the Baseline was invariably associated with lack of s/LFM appearance (**Fig 4E**). Overall, volatility and *R* values for any position correlated well, and considerably better than their correlation with *D* (**Fig 4F–4H**).

We compared the performance of the three variables (volatility, *D* and *R*) to predict the emergence of s/LFMs using a univariate logistic regression model. Higher classification metrics were observed for volatility and *R* relative to *D*, with area under the receiver operating characteristic curve (AUC) values greater than 0.9 (**Fig 4I**). By comparison, precision of these variables was low, at 0.3 for volatility and 0.24 for *R*, indicating a relatively high false-positive rate. As expected, the use of permuted networks to calculate *R* values for all spike positions resulted in a significant decline in predictive performance of this variable (**S6E Fig**). Taken together, these findings show that the emergence of s/LFMs at any spike position is associated with a state of high volatility in the ancestral lineage, as well as high volatility at adjacent positions on the protein and at associated sites on the co-variability network. While a strong relationship was observed between volatility and *R* values, as shown below, these variables exhibit different levels of predictive performance when small population sizes are used.

### Volatility profiles among sequences from the early pandemic forecast the mutational patterns of the emergent lineages

**Fig 5:**
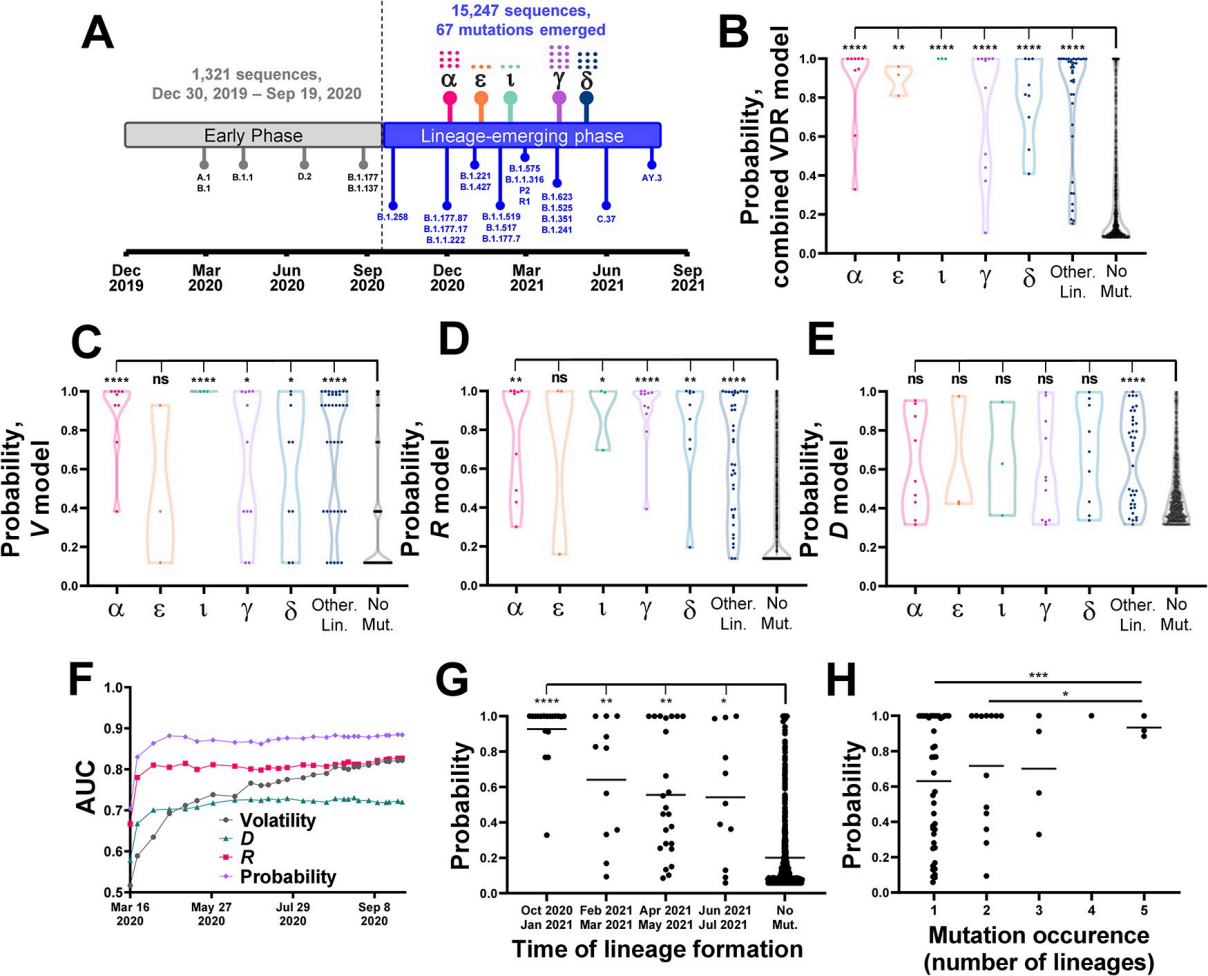
Volatility patterns among early-pandemic isolates predict emergence of mutations during the lineage-emerging phase. **(A)** Timeline for emergence of SARS-CoV-2 lineages until July 2021. Lineage emergence time is determined by the date on which 26 sequences that contain all the lineage-defining mutations were identified. Lineages with WHO variant designations are indicated by their symbols (see **S2 Table**) and the number of mutations in each is shown by dots. **(B)** Volatility, *D* and *R* values were calculated for all spike positions using the early phase sequences and applied to a logistic regression model to predict emergence of the lineage-defining mutations. Datapoints describe probabilities assigned to all spike positions and are grouped by the lineage in which they emerged. Values are compared between the mutation sites in the indicated VOCs (or minor lineages, labeled “Other Lin.”) and the no-mutation sites (“No mut”) using an unpaired T test. **(C-E)** Volatility, *D* or *R* values for all spike positions were analyzed using a univariate logistic regression model. Probability values of all sites are compared between lineages, as described above. **(F)** Volatility, *D* and *R* values and the combined probability were calculated using different amounts of time-indexed sequences from the early phase (at 50-sequence increments). AUC values are shown for predicting emergence of the 67 lineage-defining mutations in the lineage-emerging phase. **(G)** The 67 sites of mutation were grouped by the emergence time of the first lineage that contains them. Mutation probabilities assigned to the sites by sequences collected until April 1^st^ 2020 are compared with the probabilities assigned to the no-mutation sites. **(H)** Probabilities assigned by the April 1^st^ 2020 dataset are shown for mutation sites that appeared in one or more lineages. Values are compared between all groups using an unpaired T test.

We examined whether a combination of the volatility-based variables would better capture the observed evolutionary path of the virus than each of them separately. To this end, we indexed all sequences by the time of sample collection and tested if viruses that temporally preceded emergence of SARS-CoV-2 lineages can predict the mutations that they contain. For these analyses, sequences were classified by their Pango lineage designations rather than our phylogeny-based group definitions. We first determined the formation time of each lineage, defined as the date by which 26 unique sequences from the lineage were detected (see **Fig 5A** and **S2 Table**). Based on these timelines, we divided the sequences into an “early-phase” group that was used to predict emergence of lineage-defining mutations in the “lineage-emerging phase”. The early-phase group included one sequence from lineage B.1.1.7, which was removed, and none from other emergent lineages. Six minor lineages emerged early in the pandemic that contained mutations at positions 614, 222 and 477 (see **S2 Table**). To avoid a potential bias, these positions were excluded from our analyses. A total of 67 lineage-defining mutation sites were identified in the lineage-emerging phase.

The early-phase sequences were divided into 27 clusters of 50 sequences, which were used to calculate volatility, *D* and *R* values. These values were applied to a logistic regression model that was trained to predict the emergence of lineage-defining mutations using the phylogeny-indexed Baseline sequences (see Methods section). The output of the model is the probability of each site to emerge with mutations in the lineage-emerging phase. For all VOCs tested, as well as the non-VOC lineages (analyzed collectively), the probabilities calculated for the mutation sites were significantly higher than probabilities for the no-mutation sites (**Fig 5B**). Predictions based on the combined model performed better than those based on the individual variables (**Fig 5C–5E**).

To examine the changes in probabilities assigned to the sites of mutation during the early stages of the pandemic, we calculated volatility, *D* and *R* values and the combined probability using increasing numbers of sequences arranged by the time of sample collection. Interestingly, the pattern of mutations was predicted well by the time three clusters were formed (i.e., 150 unique sequences), corresponding to samples collected until April 1^st^, 2020 (**Fig 5F**). Of the individual predictors, *R* exhibited the highest performance, modestly lower than the combined probability, whereas performance of the volatility variable gradually increased. Thus, while volatility and *R* values calculated using all Baseline sequences correlated well (**Fig 4F**), the performance of *R* was considerably higher when small numbers of sequences were used (**Fig 5F**). Further analysis using the first 150 sequences showed that higher probabilities were assigned to mutation sites of lineages that emerged at earlier stages of the pandemic (**Fig 5G**). Higher probabilities were also assigned to convergent sites (i.e., those that emerged with mutations in multiple lineages, **Fig 5H**).

Taken together, these findings show that a high level of volatility at any site and at its spatial- and network-associated sites precedes emergence of mutations in descendant lineages. A small number of sequences is required to accurately estimate the likelihood of sites for emergence with lineage-defining mutations. Total volatility at network-associated sites exhibits higher sensitivity at earlier stages of the pandemic than volatility values of the sites.

### Spike mutations that define emerging VOC sublineages are accurately forecasted by patterns of volatility in their parental lineages

**Fig 6:**
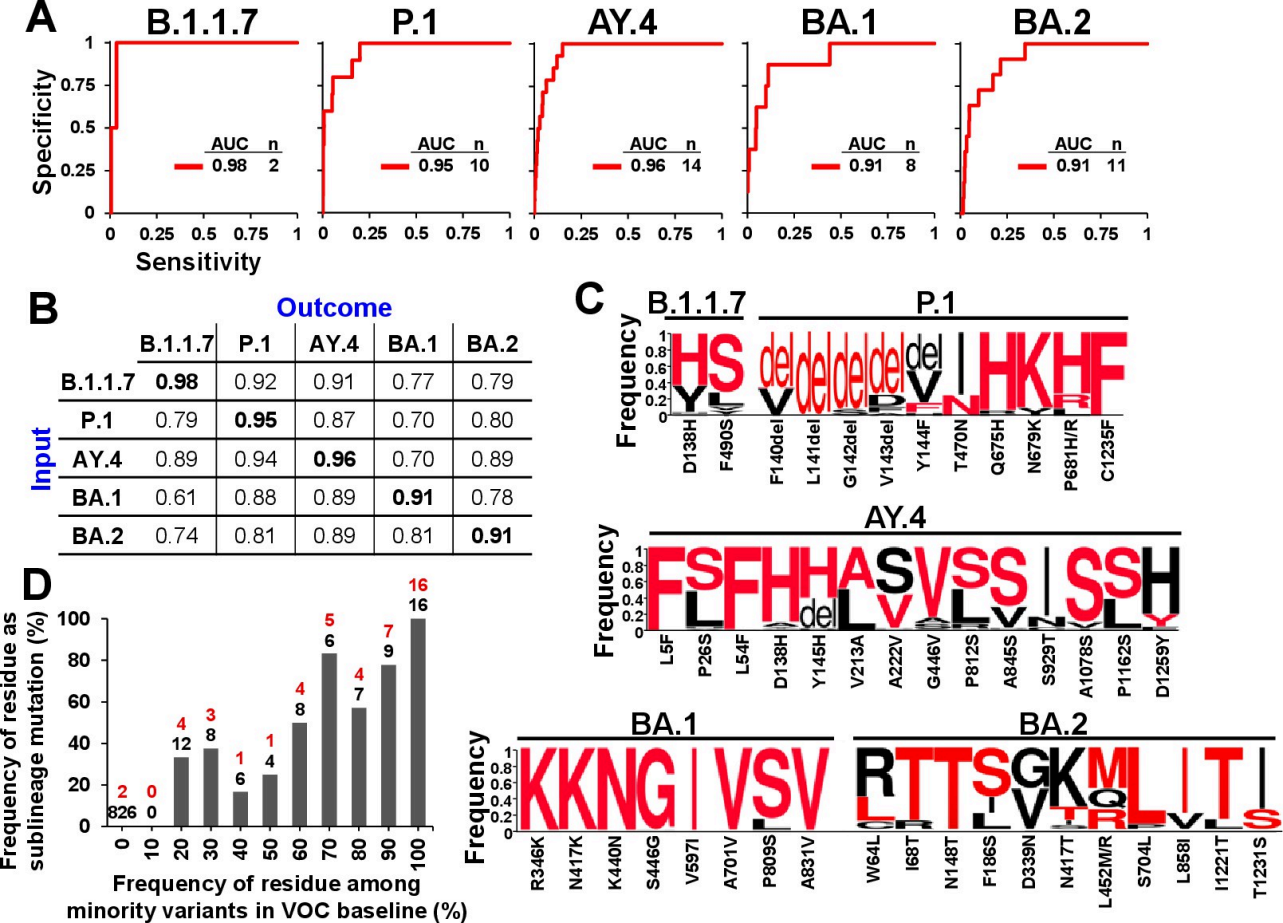
The mutational profiles of new SARS-CoV-2 sublineages are predicted well by volatility patterns in their parental lineages. **(A)** Sequences from the baselines of the indicated VOCs were used to calculate the combined probabilities for mutations at all spike positions. These values were compared with the absence or presence of a mutation that defines a Pango sublineage at the sites. The number of sublineage founder mutations (n) in each VOC and the AUC values are shown. **(B)** Mutation probabilities calculated using the sequences of each lineage (input) are compared with the sublineage mutational outcomes observed in all VOCs (outcome). The highest AUC for each lineage outcome is bolded. **(C)** Weblogos of the minority variants at sublineage mutation sites. Frequencies are expressed as a fraction of all sequences with a non-lineage ancestral residue. The residue change from the lineage ancestor is shown below the axis, and the emergent residue is also highlighted in red font. **(D)** Relationship between sampling of residues in the parental lineage and their emergence as the sublineage-defining mutations. The frequencies of all possible residues (excluding the VOC ancestral form) at the sites shown in panel C were calculated as a fraction of all minority variants identified in each VOC. The values were partitioned into the indicated bins. For example, position 138 in lineage B.1.1.7 contained the minority variants His, Tyr, Asn, Ala and Gly at 62.8, 32.7, 1.9, 1.3 and 1.3 percent, respectively, and no representation of all other residues–all 21 residue options (including N-linked glycosylation motifs and deletion events but excluding the majority variant) were distributed into their corresponding frequency bins. For each bin, we calculated the number of residues that emerged as the new sublineage-defining mutation (indicated in red font) as a percent of all instances in that bin (in black font).

Having established the model to forecast the mutations that emerged from the SARS-CoV-2 Baseline, we tested its performance to forecast the mutational profiles of sublineages that emerged from the VOCs (shown in **Fig 1A**). Sequences from the baselines of VOCs B.1.1.7, P.1, AY.4, BA.1 and BA.2 were used to forecast the mutations that define their descendent sublineages. All emergent sublineages with Pango designations and all clusters of 50 sequences that contain a non-lineage-ancestral residue as the majority variant at any site were excluded from the datasets. The remaining clusters were used to calculate volatility, *D* and *R* values and to assign a combined mutation probability to each position. As shown in **Fig 6A**, the mutations that define sublineages of the above VOCs were predicted well using the baseline sequences of each variant (see **S3 Table** for probability values). To determine lineage specificity of the predictions, we compared the probabilities assigned to all sites of each lineage with the mutational outcomes in all other lineages. Consistent with the above findings, the highest AUC values were observed for predictions of the changes that occurred in the homologous lineage (**Fig 6B**).

We also examined whether the identity of the emergent substitutions can be estimated from the amino acid profile in the parental lineage baseline. As expected of a random sampling process, in most cases, the highest frequency variant in the parental lineage appeared as the defining mutation of the emergent sublineage (**Fig 6C**). Indeed, the probability of any residue to emerge as the sublineage-dominant form was directly proportional to its sampling frequency (relative to all minority variants) in the parental lineage (**Fig 6D**). Therefore, both the site of mutation and the emergent residue are estimated well by the patterns of low frequency variants in the parental lineage.

### Mutations that imparted resistance to antibody therapeutics are predicted well by volatility patterns in the parental lineages

**Fig 7:**
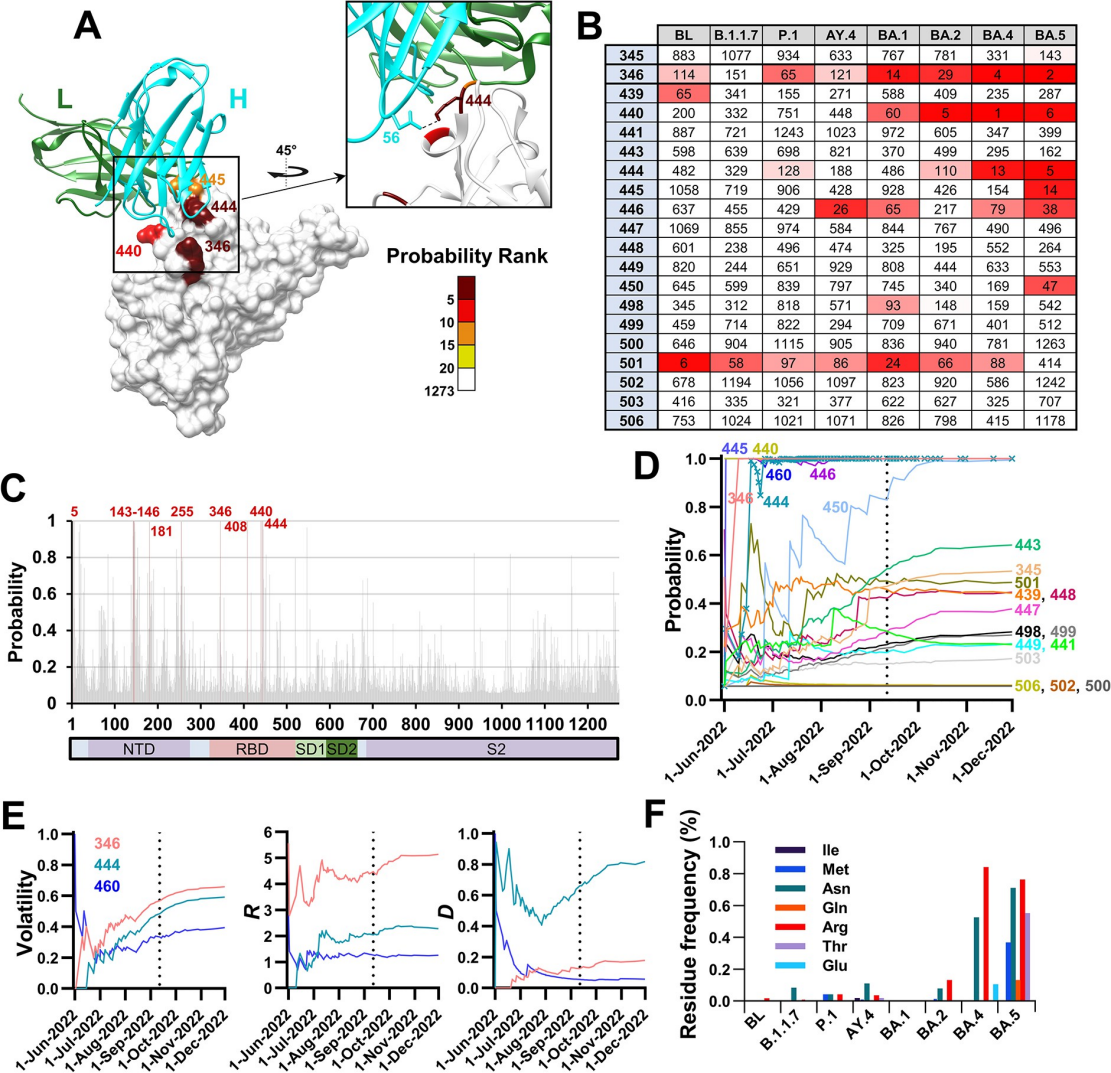
Mutations that conferred resistance to Bebtelovimab in subvariants BQ.1/BQ.1.1 are predicted well by volatility patterns in the parental BA.5 lineage. **(A)** Mutation probabilities were calculated for all positions of spike using the baseline sequences of BA.5. Residues on the structure of the RBD in complex with Bebtelovimab (PDB ID 7MMO) are colored by their probability ranks as indicated. Ranking is relative to all 1273 positions of spike whereby lower numbers indicate higher probabilities for mutations. The heavy (H) and light (L) chains are shown. **(B)** Probability ranks at the contact sites of Bebtelovimab, as calculated using the baseline sequences of the indicated lineages. **(C)** Probability values calculated for all positions of spike using the BA.5 baseline sequences. Positions with a probability value of 0.99 or greater are highlighted in red. **(D)** Sequences from the BA.5 baseline were indexed by their date of collection, divided into 50-sequence clusters, and mutation probability values were calculated for groups of increasing cluster numbers. Values are shown for the Bebtelovimab contact sites. A vertical dashed line indicates the collection date of the first BQ.1 sequence. **(E)** Changes in values of the volatility-based variables for the three sites of mutation that define subvariants BQ.1/BQ.1.1. **(F)** Frequency of minority variants at position 444, expressed as a percent of all sequences in the baseline groups of the indicated lineages.

Therapeutic antibodies against spike have been extensively used to treat COVID-19 patients at risk for progressing to severe disease [34]. The monoclonal antibody Bebtelovimab (LY-CoV-1404) was shown to reduce disease severity [35], and retained its efficacy against the BA.1 and BA.2 variants that appeared in late 2021 [36–38]. Accordingly, in February 2022, the U.S. Food and Drug Administration (FDA) issued an Emergency Use Authorization (EUA) for treatment of high-risk patients by Bebtelovimab [39]. However, in November 2022, the EUA for this antibody was revoked due to emergence of lineages BQ.1 and BQ.1.1, which are subvariants of VOC BA.5 [40]. These subvariants contain three mutations in spike relative to their parental lineage, all located in the RBD (R346T, K444T and N460K, **Fig 7A**). While positions 346 and 444 were identified as Bebtelovimab contact sites [41], the K444T change was responsible for the dramatic reduction in sensitivity [42], likely due to the loss of a salt bridge with D56 of the antibody heavy chain (**Fig 7A**) [35].

We examined the probabilities assigned by the combined VDR model to all Bebtelovimab contact sites in the different VOCs, including sequences from the baselines of lineages BA.4 and BA.5 (950 and 3,800 unique sequences, respectively). The founders of BA.4 and BA.5 contain the same amino acid sequence for spike. Both positions 346 and 444 were assigned probability values greater than 0.999 in BA.5, ranking within the top five of all 1273 spike positions (**Figs 7B, 7C,** and **S7A**). High probabilities were also assigned to these positions by the BA.4 baseline sequences. Mutation probabilities for positions 346 and 444 rapidly increased upon emergence of BA.5, reaching maximal values by mid-June 2022, more than 2 months before emergence of BQ.1/BQ.1.1 (**Fig 7D**). Similar to the profiles observed for the early pandemic viruses (**Fig 5F**), *R* values for positions 346 and 444 reached near maximal levels at earlier time points after emergence of BA.5 than the corresponding volatility values (**Fig 7E**). The high mutation probability of position 444 was also attributed to a high volatility value (**Fig 7E**). Indeed, relative to other lineages, BA.4 and BA.5 sampled a wide range of residues with diverse chemical properties at position 444 (**Fig 7F**).

We also examined the resistance that emerged to AstraZeneca’s Evusheld. This therapeutic is composed of two monoclonal antibodies—Cilgavimab and Tixagevimab, which target non-overlapping epitopes in the RBD [43]. In December 2021, the FDA issued an EUA for Evusheld as pre-exposure prophylaxis to prevent COVID-19 in immunocompromised individuals [44]. On October 5^th^ 2022, it issued a warning that Evusheld likely does not effectively neutralize a newly emergent variant, BA.4.6, a sublineage of BA.4 [45]. More than a month later, it was reported that lineages BA.2.75.2, BA.5.2.6, BQ.1/BQ.1.1 and BF.7 may also be resistant to this therapeutic [14]. Consequently, in January 2023, based on the distribution of variants circulating at the time, the FDA revoked the EUA for use of Evusheld in the United States [46].

**Fig 8:**
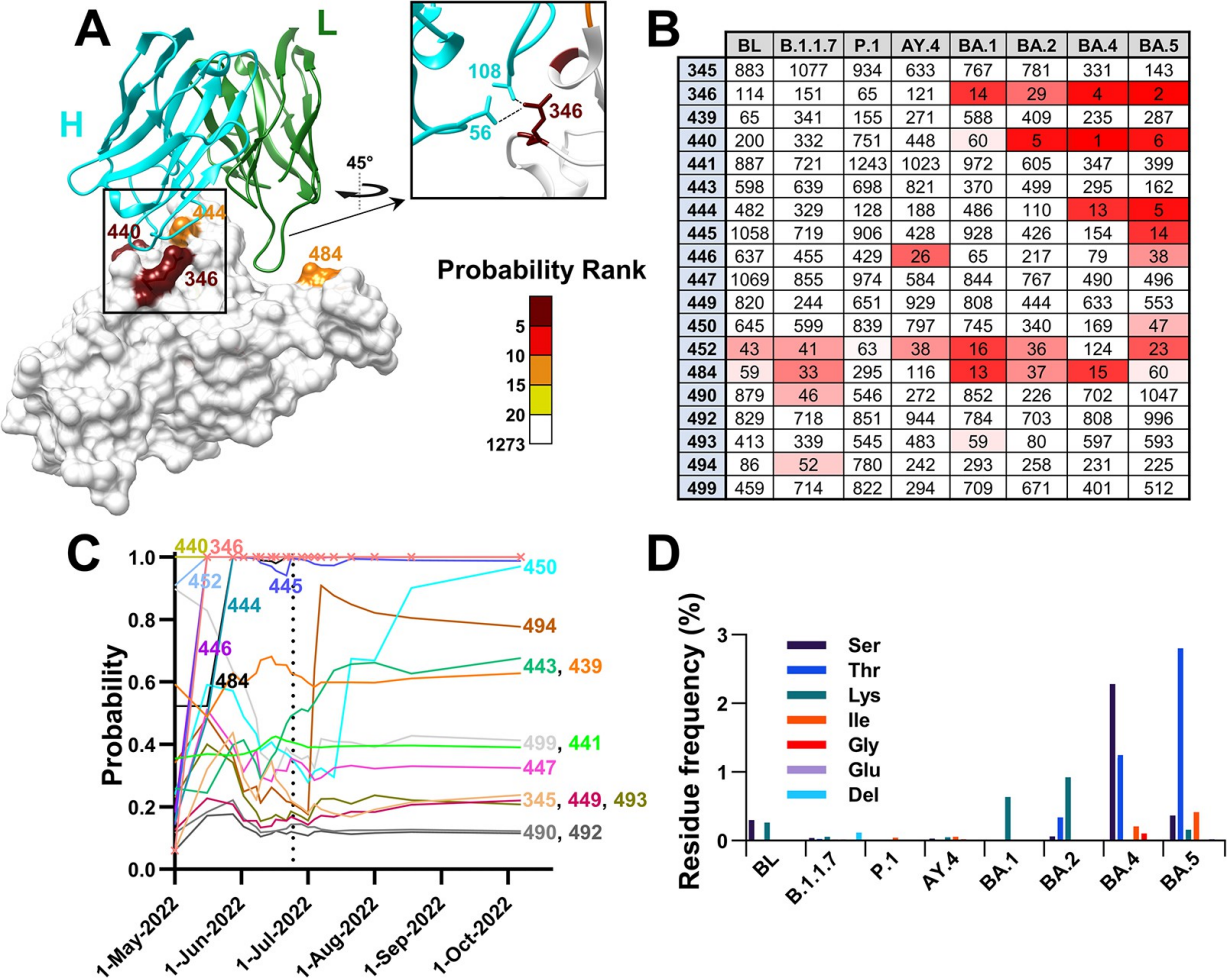
The mutation in subvariant BA.4.6 that conferred resistance to Cilgavimab is predicted well by volatility patterns in the parental BA.4 lineage. **(A)** Mutation probabilities were calculated for all positions of spike using the baseline sequences of BA.4. Residues on the structure of the RBD in complex with Cilgavimab (PDB ID 7L7E) are colored by their probability ranks as indicated. **(B)** Probability ranks for contact sites of Cilgavimab, as calculated using the baseline sequences of the indicated lineages. **(C)** Sequences from the BA.4 baseline were indexed by their date of collection, divided into 50-sequence clusters, and mutation probability values were calculated for groups of increasing cluster numbers. Values are shown for all Cilgavimab contact sites. A vertical dashed line indicates the collection date of the first BA.4.6 sequence **(D)** Frequency of minority variants at position 346, expressed as a percent of all sequences in the baseline groups of the indicated lineages. Del, deletion event.

We tested the performance of our model to forecast emergence of BA.4.6, the first subvariant that showed resistance to Evusheld. Relative to BA.4, it contained an Arg to Thr mutation at position 346 of the RBD (**Fig 8A**). While BA.4 was resistant to Tixagevimab, it retained sensitivity to Cilgavimab (and thus overall efficacy of Evusheld). Nevertheless, the R346T mutation in BA.4.6 imparted resistance Cilgavimab and was responsible for an increase in resistance to the Evusheld combination by approximately 100 fold relative to the parental BA.4 virus [45]. Calculation of mutation probabilities using the VDR model revealed that position 346 ranked fourth among all spike positions in BA.4 (see Cilgavimab contact sites in **Fig 8B** and probabilities in **S7B Fig**). Similar to position 444 (**Fig 7F**), position 346 exhibited a wide range of substitution types, including hydrophobic residues and deletion events (**Fig 8C**). Interestingly, Ser (rather than Thr) was the most frequent substitution in the baseline of BA.4, suggesting that the high volatility does not represent “contamination” of the parental dataset by BA.4.6 sequences.

We note that for all early pandemic VOCs, mutation probabilities at contact sites of Cilgavimab and Bebtelovimab were considerably lower than for VOCs BA.4 and BA.5, whereas VOCs BA.1 and BA.2 represent intermediates between the above states (**Figs 7B** and **8B**). These findings are consistent with the lineage specific probabilities for mutations at spike positions (**Fig 6B**) and the diverging nature of the evolutionary space of spike from the SARS-CoV-2 ancestral state (**Fig 1G**). Taken together, these findings show that the likelihood for emergence of mutations at clinically significant sites is forecasted well by the patterns of volatility across spike. Sequences from samples collected shortly after emergence of each parental lineage provide information that can be used to infer the evolutionary space available for each variant.

## Discussion

Since January 2022, Omicron variants and recombinants have dominated the landscape of VOCs circulating worldwide. They contain mutations in spike that reduce virus sensitivity to immune sera and COVID-19 therapeutics [36,47]. To address their unique antigenic properties [48,49], immunization protocols have been modified to include the spike protein of BA.4/BA.5 [50–52]. Nevertheless, new sublineages of Omicron lineages have emerged with mutations in spike that further impact virus transmissibility and sensitivity to COVID-19 vaccines [8,9]. Interestingly, most changes that give rise to new sublineages, did not, in our hands, show clear evidence for positive selection pressures using dN/dS-based approaches such as SLAC and MEME (**Figs 1A** and **S1B**). If evolutionary neutrality is assumed for these sites, it would be expected that the evolutionary space available for spike will be large and driven by the stochastic nature of the mutations. Here we introduce a simple probabilistic measure of the evolutionary space of the virus and show that it is distinct for each SARS-CoV-2 lineage. The model we describe reveals the protein locations where variation can be tolerated, which includes sites where mutations with large selective impact may, and do, occur.

The major finding of this work is that the mutational outcome of each site is forecasted by the variability profiles of heterologous sites on spike, and mainly by the primary nodes on the network of co-variable positions. The *R* variable is sensitive to the changes at early time points after emergence of the parental lineage, reaching maximal or near-maximal values during the first few weeks whereas volatility values gradually increase over the course of months (**Figs 5F** and **7E**). How does volatility of the “environment” capture the mutability of each site? Clustering of volatile sites on the linear sequence of the protein can be explained by mutational hotspots due to properties of the viral RNA [53,54]. Clustering on the three-dimensional structure can be explained by high permissiveness of the domain for changes due to limited impact on fitness [55]. Furthermore, adjacent residues on viral and non-viral proteins often exhibit correlated mutability and evolution [56–58]. By contrast, the association between volatility of sites separated by larger distances on the protein is less intuitive. We propose that such associations may describe the epistasis network of spike (i.e., the sites that the amino acid occupancy of one affects the fitness of another). Indeed, a recent study by Starr and colleagues demonstrated the epistatic shifts in spike that occur due to some mutations (i.e., changes in fitness profile of a site due to a mutation at a heterologous site) [59]. Such effects may be responsible for the diversifying nature of the mutational space of the protein (**Fig 1G**) and, consequently, its evolutionary space (resulting in different likelihoods for mutations at many sites in the different VOCs). The notion of lineage-specific fitness profiles was supported by the observed relationship, for many domains of spike, between volatility and the within-host variability in amino acid sequence in COVID-19 patients (**S8 Fig**). Comparison of co-variability network structure with structure of the epistasis network of spike will allow us to address this question, and may identify the adaptive sites required to facilitate changes at positions of the protein that would otherwise negatively affect virus fitness [55,60].

The model described here can be applied for two main purposes. First, as a tool for risk assessment, to determine the likelihood that resistance may emerge to each agent among currently circulating variants. Our results, which demonstrate clear antecedent warning signs for the emergence of Bebtelovimab resistance mutations in BA.5 and of Cilgavimab resistance in BA.4, suggest that therapeutics can be tailored to the evolutionary space of each lineage. This space can be inferred at early stages after emergence of the variant in the population. We also show that the time horizon for the predictions is long, with a gradual decline in performance over the course of 9 months from the early phase of the COVID-19 pandemic (**Fig 5G**). In addition, the high performance of the model was retained across multiple generations of subvariants (i.e., to forecast the mutational patterns of increasing-order generations of the VOCs, **S9 Fig**). Consistent with these results, a modest decline in performance of the model was observed for predicting emergence of sLFMs relative to LFMs from the Baseline group. The volatility, *R* and *D* metrics were all modestly lower for the sites of sLFMs relative to the LFMs (**Figs 2C, 3F,** and **4D**). Nevertheless, both mutation types were forecasted well by the combined VDR model, with AUC values of 0.937 and 0.904 for the sites of LFMs and sLFMs, respectively. Thus, while new subvariants continuously emerge, these findings indicate the utility of the model through time as well as multiple generations of subvariants. Such advance notice of the imminent changes in each lineage allows us to test their impact on virus sensitivity to candidate therapeutics and, given multiple candidate agents [61], can guide selection of those with the lowest likelihood for emergence of resistance.

The model can also be applied to map the evolutionary space of a new VOC that may emerge in the future, during the initial stages of its spread when only a limited number of sequences are available. Different variables exhibit different levels of performance at different stages after emergence of the new variant. For example, while the volatility variable contributes most when ample data is available, *R* exhibits near-maximal sensitivity when only a small number of sequences are applied (**Figs 5F** and **7E**). Such differences highlight the need to tailor prediction models to the amount of data available at each time point. The ability to map the evolutionary space of the protein at very early stages, when other tools may not be effective, can contribute to preparedness for new phenotypic subvariants that may emerge. Our prediction model was designed to address such cases of newly emergent variants, when several divergent forms may appear (such as the early stages of the COVID-19 pandemic, when both 614D and 614G were detected). Accordingly, we defined s/LFMs as substitutions relative to the ancestral sequence of the parental lineage (rather than the consensus) since it does not assume that the dominant form in the new lineage baseline will also appear in any emergent sublineage. Based on this definition, position 614 was designated as an LFM that emerged from the SARS-CoV-2 Baseline despite the clear dominance of the 614G form in the SARS-CoV-2 Baseline Group. To determine the impact of this choice, we examined performance of the prediction model with and without position 614 designated as an LFM. Sequences from the Baseline Groups were used to predict the s/LFMs in the Terminal Groups. Nearly identical coefficients were assigned by the logistic regression model to Volatility, *D* and *R*, with similar AUC values (0.936 and 0.930 for the tests with and without position 614 as an LFM, respectively). Therefore, the model used to forecast emergence of mutations in sublineages of the VOCs was not impacted by selecting the lineage ancestral sequence to define mutations.

A primary concern in analysis of the dynamic population of SARS-CoV-2 sequences are misclassification events of the sequences. Such events may result in contamination of the baseline group (used for calculating volatility) by sequences from the emergent sublineages, potentially leading to spuriously high volatility at the sites of sublineage mutations. To avert such effects, we apply three approaches to classify the sequences: **(i)** Based on their Pango lineage designations, determined by the entire SARS-CoV-2 genome **(ii)** Based on our phylogenetic analysis of the spike nucleotide sequences, and **(iii)** By collection dates of the samples. The use of multiple classification approaches as well as manual inspection and curation of the datasets reduces the risk for “contamination” of the VOC baselines by misclassified sublineage sequences or by potential founder variants of the sublineages. Importantly, the dominant contribution of the *R* variable to the prediction (e.g., **Fig 5F**), which is based on heterologous sites on spike, supports the notion that the predictive capacity of the model is not impacted by the above types of events.

We note that, despite the high predictive performance shown, these studies constitute a relatively simple framework to identify variables associated with the changes in SARS-CoV-2 spike. Higher performance is likely to be achieved by incorporating additional predictors, including selection pressures applied on the sites [62], phylogenetic tools to identify convergent and high mutability sites [27,60], *in vitro* fitness profiles [55], relative prevalence of circulating variants and their rates of spread [10], nucleotide-level substitution models [63] and patterns of mutations within the host [64–66]. Furthermore, from a computational perspective, our strategy can be refined by applying alternative methods to define the architecture of the co-variability network, by using more sophisticated learners to combine the volatility-based variables, and by applying models that are tailored to the properties of each domain or position of the protein. Finally, the use of more homogenous donor populations, divided by their infection and vaccination status, will allow us to account for the effects of the immune response on the evolutionary path of each variant.

## Methods

### Sequence alignment

Nucleotide sequences of SARS-CoV-2 isolated from humans were downloaded from the National Center for Biotechnology Information (NCBI) database, the Virus Pathogen Database and Analysis Resource (ViPR) and from the GISAID repository [67]. The following processing steps and analyses were performed within the Galaxy web platform [68]. First, excess bases were trimmed using *Cutadapt*, using 5’-ATGTTTGTT-3’ and 3’-TACACATAA-5 “adapters” that flank the spike gene. Adapter sequences were allowed to match once with a minimum overlap of 5 bases, an error rate of 0.2 with a sequence length between 3,700 and 3,900 bases. All sequences with any nucleotide ambiguities were removed by replacing the non-standard bases with ‘N’ using *snippy-clean_full_aln*, followed by filtration of N-containing sequences using *Filter FASTA*. Sequences that cause frameshift mutations were excluded using *Transeq*. Nucleotide sequences were aligned by *MAFFT*, using the FFT-NS-2 method [69]. The aligned sequences were then “compressed” using *Unique*.*seqs* to obtain a single representative for each unique nucleotide sequence [70]. Nucleotide sequences were then translated with *Transeq* and aligned with *MAFFT*, FFT-NS-2 [69]. The first position of each PNGS motif triplet (Asn-X-Ser/Thr, where X is any amino acid except Pro) was assigned a distinct identifier from Asn. All phylogenetic analyses were performed using the full-length spike protein, which included several sequences with amino acid insertions. To maintain consistent numbering of spike positions, all calculations described in this work were performed for the 1,273 positions of the spike protein in the SARS-CoV-2 reference strain (accession number NC_045512).

### Phylogenetic tree construction and analyses

A maximum-likelihood tree was constructed for the aligned compressed nucleotide sequences using the generalized time-reversible model with CAT approximation (GTR-CAT) nucleotide evolution model with *FASTTREE* [71]. The tree was rooted to the sequence of the SARS-CoV-2 reference strain with MEGAX [72]. To divide the tree into “Groups” of sequences, we used an in-house code in Python (see link to GitHub repository in the Data Availability). This tool uses the Newick file to divide the dataset into sequence Groups with a user-defined genetic distance between their centroids. For all analyses we used a distance of 0.004 nucleotide substitutions per site for Group partitioning. Groups that did not contain at least 50 unique sequences were excluded. To distinguish between Baseline groups and Terminal Groups, we used a distance of 0.0015 nucleotide substitutions per site between each group centroid and the SARS-CoV-2 reference strain.

### Calculations of volatility

To calculate volatility of spike positions, we divided all sequences of each group into clusters of 50 sequences. Sequence variability in each cluster was quantified using two approaches. To calculate volatility values, we used a binary approach, whereby each position in a 50-sequence cluster was assigned a value of 1 if it contains any diversity in amino acid sequence, or a value of 0 if all sequences in the cluster contain the same amino acid. Thus, each cluster is assigned a 1,273-feature vector that describes the absence or presence of volatility at each position of spike. Volatility was then calculated by averaging values by position across all clusters. For calculations of *D* or *R* values for each position *i*, we used a quantitative approach to define volatility at positions associated with *i* (i.e., at positions *s* and *q* in **Eq 1** and **Eq** 2, respectively). Briefly, sequence variability within each cluster was measured by assigning distinct hydropathy scores to each amino acid according to a modified Black and Mould scale [73]. The Asn residue of PNGS motifs and deletions were also assigned unique values. The values assigned were: PNGS, 0; Arg, 0.167; Asp, 0.19; Glu, 0.203; His, 0.304; Asn, 0.363; Gln, 0.376; Lys, 0.403; Ser, 0.466; Thr, 0.542; Gly, 0.584; Ala, 0.68; Cys, 0.733; Pro, 0.759; Met, 0.782; Val, 0.854; Trp, 0.898; Tyr, 0.9; Leu, 0.953; Ile, 0.958; Phe, 1; deletion site, 1.5. Variability in each cluster was calculated as the standard deviation in hydropathy values among the 50 sequences, and variability values of all clusters were averaged to obtain the volatility value for each position *s* or *q* (i.e., *V*_s_ or *V*_q_, respectively).

### Autocorrelation analyses

To quantify the clustering of volatility on the linear sequence, we tested its autocorrelation using lags of 1 to 10 positions. The autocorrelation coefficient was calculated as:

$$r(k)=\left(\frac{1}{n}\right)\sum_{i=1}^{n-k}\left[(V_{i}-\mu )(V_{i+k}-\mu \right)]$$

where *r*(*k*) is the autocorrelation coefficient at lag *k*, *n* is the number of positions in the spike domain tested, *V*_*i*_ and *V*_*i*+*k*_ are the volatilities at positions i and i+*k*, respectively, and *μ* is the mean volatility at all positions in the domain tested. To calculate statistical significance of the lagged correlations, we assumed independence of the volatility values at positions i and i+*k*, and used the Spearman rank correlation test. P-values for a two-tailed were reported.

### Lineage specificity of spike volatility profiles

To determine relationships between volatility profiles of spike in the diverse lineages, we partitioned each lineage into 10-cluster groups (500 sequences). All sublineages with Pango designations and all 50-sequence clusters with a dominant non-lineage-ancestral residue at any spike position were removed from the datasets. Within each group, the absence or presence of amino acid variability at each spike position was determined. All groups were thus assigned 1273-bit strings that describe the absence (0) or presence (1) of volatility at each position of the protein. Jaccard distances between the strings were calculated and all groups compared using the Unweighted Pair Group Method with Arithmetic Mean (UPGMA) method [24, 25]. The output (in Newick format) was used to generate a dendrogram plot with MEGAX.

To determine the lineage specificity of the volatility patterns, we used a modification of an approach we previously described [26]. Briefly, each SARS-CoV-2 lineage was divided into groups of 10 clusters (500 sequences). Volatility in the groups was calculated for all positions of spike, and each group assigned a 1273-feature vector that describes the level of volatility at all positions of spike. To compare the vectors, we first calculated for each lineage *L* the coordinates of the centroid (*C*_L_) among vectors from the same lineage. The mean intra-lineage distance (*d*_intralineage_) was calculated as the average Euclidean distance between the lineage centroid *C*_*L*_ and all groups from the same lineage *G*_*L*_, formally $\bar{dist(C_{L},G_{L})}$. In addition, we calculated the mean inter-lineage distance (*d*_interlineage_) as the average Euclidean distance between the centroid of lineage *L* and all other lineage centroids $\bar{dist(C_{L},C_{L})}$ ∀L’≠L. We define the ratio as: $ratio=\frac{d_{intralineage}}{d_{interlineage}}$. The baseline ratio (*S*_base_) was calculated as the *ratio* using the non-permuted data. Under the null assumption concerning the evolution of volatility profiles, the intra-lineage distances are expected to be comparable to the inter-lineage distances, while under the lineage-specific alternative, we expect clustering of volatility profiles within each lineage even across distinct 10-cluster groups. To test this, lineage identifiers were permuted and randomly assigned to each group, from which the permuted ratio (*S*_rand_) was calculated. The permutation process was repeated 10,000 times. The P value was calculated as the fraction of the 10,000 tests that *S*_rand_ was smaller than *S*_base_.

### Co-variability network construction

To determine the co-variability of any two spike positions, we generated a matrix that contains binary volatility values in all clusters of the tested group (rows) for all 1,273 spike positions (columns). Co-occurrence of a volatile state at any two positions was calculated using Fisher’s exact test and the associated P-value was determined. To construct the network of co-variability, we used as input the matrix that describes the -log_10_(P-value) between the volatility profiles of any two spike positions, whereby nodes are the positions of spike and the edges that connect them reflect the P-values of their association. Network structure was visualized using the open-source software *Gephi* [74]. Networks were generated using different P-value thresholds (i.e., an edge was assigned only if the P-value was lower than 0.1, 0.05 or 0.01). To determine robustness of network structure, we randomly deleted 10, 20 or 30 percent of all edges for each of the networks, and network topological properties were computed using the *Cytoscape Network Analyzer* tool [75]. Two metrics were calculated for the complete and depleted networks: **(i)** Degree distribution, and **(ii)** Closeness centrality [32].

To generate random networks, we applied as input the data that describe absence or presence of volatility at all spike positions in all 114 clusters of the BL group. For each position separately, cluster labels were permuted, and the new volatility profile was used to calculate P-values using Fisher’s test and to construct a network, as described above.

### Calculations of selection pressures

To infer the selective pressures applied on SARS-CoV-2 spike positions we used the Single-Likelihood Ancestor Counting (SLAC) method [18]. This counting- and maximum likelihood-based approach estimates the rates of synonymous (S) and nonsynonymous (N) substitutions assuming that selection pressures applied on the positions are constant [18]. While SLAC is commonly used to infer selective pressures on SARS-CoV-2 spike [11, 76–78], we also used the Mixed Effects Model of Evolution (MEME) method [79]. MEME tests the hypothesis that sites are subjected to constant positive selection across the lineage as well as episodic positive selection (i.e., on part of a lineage). For both SLAC and MEME, substitution rates were inferred for each site from the input nucleotide alignment and from the associated phylogenetic tree. The input nucleotide sequences (for each lineage separately) were aligned by *MAFFT*, using the FFT-NS-2 method [69]. The maximum likelihood tree was constructed using FASTTREE (GTR-CAT) [71]. Computations of dN and dS were conducted using the *HyPhy 2*.*5* software package [80] within the Galaxy platform [78].

### In-host amino acid variability at spike positions in COVID-19 patients

Analysis of in-host amino acid variability in deep sequencing data from COVID-19 patients was performed using samples from patients infected by SARS-CoV-2 variants Alpha (B.1.1.7), Delta (B.1.617.2) and Gamma (P.1) (33, 30 and 33 samples from each lineage, respectively) [81]. All samples were collected from COVID-19 unvaccinated individuals and sequenced by Davis and colleagues using an Illumina NovaSeq 6000 instrument with the ARTIC V3 primer set [66]. Samples were selected for their high coverage of the SARS-CoV-2 genome. Raw sequencing reads were processed using *Trimmomatic* to remove adapters using the following settings: ILLUMINACLIP:${params.adapters}:2:30:10:8:true LEADING:20 542 TRAILING:20 SLIDINGWINDOW:4:20 MINLEN:30 [82]. Low-quality reads (Phred scores lower than 30) were removed using *FASTX-Toolkit* [83]. Sequences were then mapped to the SARS-CoV-2 genome (accession number NC_045512.2) using *minimap2* [84]. Only reads that met a minimum mapping quality score of 30 and mapped to the spike gene with a minimum length of 30 bases were retained. The sequences were then codon-aligned using *bealign* [80] and subsequently translated using *Transeq*. Any sequences that resulted in an early stop codon were removed. Bases with a minimum quality score threshold of 20 were used to build a consensus sequence using *ivar consensus* [85]. The lineage association of each patient consensus was confirmed using *Nextclade* and *Pangolin* [86, 87]. To calculate the frequency of amino acids at each site, only positions with coverage greater than 100 reads were used. To minimize noise in the data, for each residue at each spike position, we calculated the mean frequency among the samples of the lowest quartile; this value was then subtracted from the readouts. The presence of amino acid variability at each position was determined by the occurrence of at least one minority amino acid with a frequency greater than 1% of all reads.

### Spatial clustering of volatility and calculations of the variable *D*

We performed a permutation test to determine the spatial clustering of volatile sites around each spike position. To this end, for each position *i*, we identified the 10 closest positions on the trimer, using coordinates of the cryo-EM structure of the cleavage-positive spike (PDB ID 6ZGI) [31]. We then calculated for each position *i* the statistic $T_{i}^{0}$:

$$T_{i}^{0}=\sum_{j\in \varphi_{i}}V_{i}^{0}*V_{j}^{0}$$ [3]

where $V_{i}^{0}$ describes the volatility at position *i*, $V_{j}^{0}$ is the volatility at the j^th^ neighboring position to *i*, and *φ*_*i*_ denotes the positions numbers of the 10 closest neighbors to position *i*. We then permuted all positions identifiers other than *i* and calculated the statistic $T_{i}^{k}$:

$$T_{i}^{k}=\sum_{j\in \varphi_{i}}V_{i}^{0}*V_{j}^{k}$$ [4]

where $V_{j}^{k}$ is the volatility at the j^th^ adjacent position in the *k*^th^ permutation (*k = 1*,*2*, *… 5*,*000*). Under the null hypothesis of no spatial clustering, we would expect the neighbor labels to be arbitrary. We therefore test this null hypothesis by estimating the probability of observing a positive departure from the null distribution via:

$$P=\frac{\sum_{k=1}^{N}I_{\left\{T_{i}^{k}\geq T_{i}^{0}\right\}}}{N}$$ [5]

where *N* is the total number of permutations (5,000) and *I* is the indicator function. Therefore, the P-value quantifies the fraction of times the volatility of the surrounding residues is larger for the permuted values relative to the non-permuted values.

To calculate *D*, we measured for each position *i* the total volatility at all sites that are within a distance of 6Å on the spike trimer structure. The coordinates of the cryo-electron microscopy structure of the cleaved spike protein in the closed conformation (PDB ID 6ZGI) were used [31]. Coordinates of all atoms were included; N-acetyl-glucosamine atoms were assigned the same position number as their associated Asn residues. We note that the 6ZGI structure is missing the following spike residues (numbered according to the SARS-CoV-2 reference strain): 1–13, 71–75, 618–632, 677–688, 941–943 and 1146–1273. To calculate *D* values for these positions, we applied the volatility values of the positions immediately adjacent on the linear sequence of spike (i.e., positions -1 and +1).

### Combined model to predict emergence of LFMs and sLFMs

To assign a probability for each position to emerge with a mutation, we used a logistic regression model that applies volatility, *D* and *R* values. The model was trained using volatility, *D* and *R* values calculated using the 5,700 sequences of the Baseline group, with the positive outcome being the 43 LFM and 16 sLFM sites described in **S1 Table**. To this end, we first created interaction terms between the initial predictors (i.e., volatility, *D* and *R*). To address the class imbalance in our datasets (59 of the 1,273 spike positions appeared with LFMs or sLFMs) we used the adaptive synthetic sampling approach (ADASYN) [88]. Nested cross-validation was used to tune the model while estimating the metrics of interest. This procedure was also used to generate the prediction probabilities for each position. Five folds were used for both the inner and outer parts of the nested cross-validation. Grid search was utilized to optimize hyperparameters with the area under the receiver operating characteristic curve as the objective for optimization. The model-specific parameters that we incorporated into the hyperparameter tuning procedure are the inverse of the regularization strength C and the penalty type. For this purpose, we used a set of values from 0.001 to 100 for parameter C, and for penalization we used L1 norm, L2 norm, elastic net, or no penalty in the parameter space. Since we used ADASYN to handle the class imbalance, we also added the number of positions with similar feature values as another hyperparameter to the search grid. The number of positions with similar feature values was set between 5 and 45. As classification metrics, we used sensitivity, specificity, precision, recall, AUC and balanced accuracy. The balanced accuracy metric, which is the average of sensitivity and specificity, was used due to the relative imbalance in the datasets.

## Supporting information

S1 Fig Selection pressures applied on spike positions that emerged with sublineage-defining mutations in diverse SARS-CoV-2 lineages. **(A)** Distribution of sample collection times for sequences of the indicated lineages and the SARS-CoV-2 baseline (BL) group. All sequences included in this study were unique, lacked any nucleotide ambiguities and appeared at least twice in the population (see numbers in parentheses). **(B)** Nucleotide sequences of isolates from the baselines of the indicated lineages were used to infer the rates of nonsynonymous and synonymous mutations by the Single-Likelihood Ancestor Counting (SLAC) method and by the Mixed Effects Model of Evolution (MEME) method. Sites assigned P-values smaller than 0.1 by either (or both) methods are colored as indicated. Black dots indicate sites of mutation that define VOC sublineages that emerged until April 8^th^ 2022.
(TIFF)

S2 Fig Calculation of volatility at spike positions in SARS-CoV-2 lineages. **(A)** Cluster size selection. To calculate volatility, we used 615,374 nucleotide sequences of the spike gene collected until July 2021 to construct a maximum likelihood tree. Only a single representative of each spike sequence that lacks ambiguities and appears at least twice was used. The Newick file was then partitioned into Groups separated by a minimal distance of 0.004 substitutions per site (see **Fig 2A** and **S1 Table**). The 20 Groups were classified as Baseline or Terminal using a threshold of 0.0015 substitutions per site between the SARS-CoV-2 ancestral sequence and the Group centroid. Each Group was then partitioned into clusters of 25, 50, 100 150 or 200 sequences. The number of sequences assigned to clusters using each partition approach is shown. **(B)** To calculate “volatility” of each spike position, we determined the proportion of clusters in each Group that exhibit any variability in amino acid sequence at the site. Volatility values for all spike positions based on partition of the Baseline Groups into 25- or 50-sequence clusters are shown. The P-value in Levene’s test of the equality of variance was smaller than 0.000001, strongly suggesting violation of the assumption of equal variance between the two populations. **(C)** Volatility calculated for all positions of spike using sequences from the indicated lineages or the SARS-CoV-2 BL group. Data represent sequences from samples collected worldwide until the following dates: BL, July 2021; P.1, AY.4, AY.4.2 and BA.1, February 2022; B.1.1.7, BA.1.1 and BA.2, March 2022. Volatility values are color-coded as indicated. **(D)** Schematic of our approach to compare volatility profiles of spike in diverse SARS-CoV-2 lineages.
(TIFF)

S3 Fig Volatility at sites of mutation in diverse VOCs and its relationship with the inferred selection pressures applied on them. **(A)** Volatility at sites of sublineage-defining mutations in the indicated VOC baselines. Positions are clustered by the lineage in which the mutations appeared (outlined by dashed black lines). Cells are colored by the relative volatility measured at the site in the different VOCs. Sites of VOC-defining mutations (M) are colored in grey. Black dots indicate lineages with significantly lower volatility than that in the lineage of mutation emergence (P-value < 0.05). Significance was calculated using Fisher’s exact test that compares variability in the 50-sequence clusters of the different lineages. **(B)** Relationship between volatility and selection pressures at spike positions in diverse VOCs. Amino acid sequences of isolates from the baselines of the indicated lineages were used to calculate volatility at all spike positions. Nucleotide sequences of these isolates were used to infer the rates of nonsynonymous and synonymous mutations by the SLAC method. To determine significance of these estimates, P-values derived from a two-tailed extended binomial distribution were calculated, and all sites with P-values larger than 0.1 were assigned a dN-dS value of 0. Data points of positions that emerged with sublineage-defining mutations until April 8^th^ 2022 are colored red.
(TIFF)

S4 Fig A high level of volatility at spike positions is associated with emergence of founder mutations in SARS-CoV-2 lineages. **(A)** Phylogenetic tree constructed from 16,808 unique spike sequences from samples collected worldwide until July 2021. Examples are shown of lineage-founder and sublineage-founder mutations in spike. (Left) Branches are colored by the amino acids that occupy spike position 501. The pattern corresponds to presence of a lineage founder mutation in G_T1_(α), G_T6_(γ) and G_T8_. (Right) Branches are colored by the amino acids that occupy position 1191, showing a sublineage-founder mutation within G_T1_(α). **(B)** Volatility of spike positions of the S2 subunit, as calculated using the baseline group of 5,700 sequences (114 clusters). Red bars indicate positions with lineage-founder mutations (in any terminal or baseline group). Blue bars indicate positions with sublineage-founder mutations. FP, fusion peptide; HR, heptad repeats; TM, transmembrane domain; CT, cytoplasmic tail. **(C)** Thirty spike positions with the highest volatility values. The baseline (“B”) or terminal (“T”) groups that contain mutations at these positions are indicated. **(D)** Classification metrics for evaluating performance of volatility calculated with different cluster sizes using univariate logistic regression. Error bars, standard errors of the means for five-fold cross-validation. Bal. Acc., Balanced Accuracy.
(TIFF)

S5 Fig Prediction of lineage founder mutation sites by volatility at adjacent positions on the linear sequence of spike. **(A,B)** Volatility values at positions of the NTD and RBD were calculated using the SARS-CoV-2 baseline group of sequences. These values are compared with the relative solvent exposure of the residues on the unliganded structure of spike (PDB ID 6ZGI). r_S_, Spearman correlation coefficient. P-values, two-tailed test. **(C)** For each position of spike, we calculated the average volatility for eight-position windows on the linear sequence, which include the -1 to -4 positions and the +1 to +4 positions (Vol(8p)). These values are compared between positions that emerged with LFMs, sLFMs and positions with no such mutations. **(D)** The number of positions that emerged with LFMs or sLFMs when the Vol(8p) value was zero or larger than zero. **(E)** Classification metrics for evaluating performance of the *D* and Vol(8p) variables to predict emergence of s/LFMs using univariate logistic regression. Error bars, standard errors of the means for five-fold cross-validation. Bal. Acc., balanced accuracy.
(TIFF)

S6 Fig Structural properties of the network of co-variable sites. **(A)** The co-occurrence of amino acid variability at any two spike positions in the 114 clusters of the SARS-CoV-2 baseline group was determined by Fisher’s exact test. The distribution of P-values is shown. **(B)** The co-variability network around position 614 as the root node. Edges were assigned to position pairs if the P-value was smaller than 0.01. First- and second-degree nodes are shown. Node size and color correspond to the number of triangle counts for each position. **(C)** Networks of co-variability between all spike positions were constructed using P-value thresholds of <0.01, <0.05 or <0.1. For each network, we randomly deleted 10%, 20% or 30% of edges and examined the effects on network stability. Closeness centrality values are shown for the intact and depleted networks. Higher values indicate shorter distances to all other nodes. Bars indicate the second and third quartiles and whiskers indicate minimum and maximum values. **(D)** Comparison of the original and permuted networks. For each position of spike separately, the binary indicators of amino variability were shuffled among the 114 clusters of the SARS-CoV-2 baseline group. The permuted data were used to construct a new co-variability network. This process was performed three times. The degree distribution for the original and permuted networks are shown. **(E)** *R* values were calculated for all spike positions using the original and three permuted networks. These values were compared with the absence or presence of a lineage founder mutation at the sites using univariate logistic regression. Classification metrics of *R* values calculated using the original and three permuted networks are shown. Error bars, standard errors of the mean for five-fold cross validation.
(TIFF)

S7 Fig Probabilities for mutations at contact sites of antibody therapeutics. Sequences from the baselines of the indicated VOCs (or the SARS-CoV-2 Baseline group, BL) were used to calculate volatility, *D* and *R* values for all positions of spike. These values were used to calculate the combined probability for mutations at each position. Probabilities for the contact sites of antibodies Bebtelovimab **(A)** and Cilgavimab **(B)** are shown. Probability ranks are shown in **Figs 7B** and **8B**.
(TIFF)

S8 Fig Volatility of spike positions corresponds well with their level of in-host variability. **(A)** Relationship between in-host amino acid variability and population-level volatility of spike positions. Samples collected from 96 unvaccinated COVID-19 patients were deep sequenced. The consensus sequences of the samples matched lineages B.1.1.7 (VOC Alpha), B.1.617.2 (Delta) and P.1 (Gamma) (33, 30 and 33 patients, respectively). For each position in each sample, we determined the absence or presence of amino acid variability, defined by presence of any residue with a frequency greater than 1% of all reads. The fraction of patients from each lineage containing amino acid variability at each spike position was then calculated. These values are compared with the population-level volatility values of the sites using the Spearman rank test. P-values for the indicated domains are shown. SD1/SD2, subdomains 1 and 2 of the S1 spike subunit. **(B)** Correlations between in-host variability and population-level volatility for the 42 sublineage mutation sites shown in **Fig 1A**. Sites of VOC-defining mutations in any lineage were excluded from the analysis.
(TIFF)

S9 Fig Performance of the combined model to predict emergence of mutations that define the n^th^-order generation of the VOCs. To examine the time horizon of the predictions, we tested performance of the model to forecast the mutational patterns of increasing-order generations. Only daughter lineages composed of 100 or more cases were considered. Recombinant sublineages were not included in the analysis. For each VOC, we calculated the combined probability for mutations at all spike positions based on the volatility in the VOC baseline. Position probabilities were compared with the mutational outcomes in all subvariants of the n^th^-order generation of the VOC (i.e., absence or presence of a sublineage-defining mutation at the site among all n^th^-generation sublineages). Performance was evaluated by the AUC metric. The number of unique sites of mutation in each generation is indicated by numbers above the bars.
(TIFF)

S1 Table Founder mutations in spike that define SARS-CoV-2 lineages and sublineages, as determined by phylogenetic partition. ^a^ Grouping is based on phylogenetic analysis of 16,808 unique nucleotide sequences of spike isolated from samples collected worldwide between December 2019 and July 2021. ^b^ Groups were assigned to the baseline set of sequences (G_B_) if their centroid was located 0.0015 or less nucleotide substitutions per site from the reference spike sequence (accession number NC_045512). ^c^ Only Pango lineages that represent 10 percent or more of sequences within a group are listed. ^d^ The number of G_T3_(δ) sequences isolated from samples collected between December 2019 and September 2021 is indicated in parentheses. ^e^ A mutation is defined as lineage founder if it is identified in the inferred ancestral sequence of the group and in more than 50 percent of group sequences. ^f^ PNGS indicates presence of Asn at the first position of a PNGS triplet where the third position is occupied by Thr or Ser and the second position is not occupied by Pro. ^g^ A mutation is defined as sublineage founder if it is not identified in the inferred group ancestor and is the dominant residue in at least one group cluster but less than 50% of all group clusters. ^h^ Sublineage-founder mutation that appeared in G_T3_(δ) between July 2021 and September 2021 are indicated in parentheses and in bold font.
(TIFF)

S2 Table Mutations in the spike protein of SARS-CoV-2 lineages that emerged until July 2021. ^a^ The date of lineage emergence is defined as the timepoint by which at least 26 sequences of the indicated lineage were detected. ^b^ Spike mutations associated with the Pango lineage based on data published from Outbreak.info, 2021. ^c^ Values in parentheses describe the probabilities assigned to each site for a mutation, based on a logistic regression model that combines volatility, *D*, and *R* values (see Fig 6B). ^d^ Sites of mutations in lineages that formed before September 19^th^ 2020 are shown in red font. These positions were excluded from our time-indexed analyses.
(TIFF)

S3 Table Mutations in spike protein of SARS-CoV-2 sublineages, as defined by the Pango classification system. ^a^ Sublineages that appeared until April 8^th^, 2022. ^b^ Reversions to the SARS-CoV-2 ancestral residues are shown in bold red font. ^c^ Unique mutations in the second-order sublineages that appeared until June 17^th^, 2022 but do not appear in the ancestral (first-order) sublineage are shown.
(TIFF)

S1 Data Raw data underlying plots and graphs in Figs 1–7. (XLSX)

S2 Data Raw data underlying plots and graphs in S1–S9 Figs. (XLSX)

S3 Data Text file containing the phylogenetic tree (in Newick format) used to generate Fig 1B. (TXT)

S4 Data Text file containing the phylogenetic tree (in Newick format) used to generate Fig 2A. The ancestral SARS-CoV-2 sequence is represented by the identifier NC045512.
(TXT)
