# Supplementary figures and images for: Mapping the Evolutionary Space of SARS-CoV-2 Variants to Anticipate Emergence of Subvariants Resistant to COVID-19 Therapeutics
Source: PLoS Comput Biol. 2024 Jun 10;20(6):e1012215. doi: 10.1371/journal.pcbi.1012215 (PMC11192331; doi:10.1371/journal.pcbi.1012215)

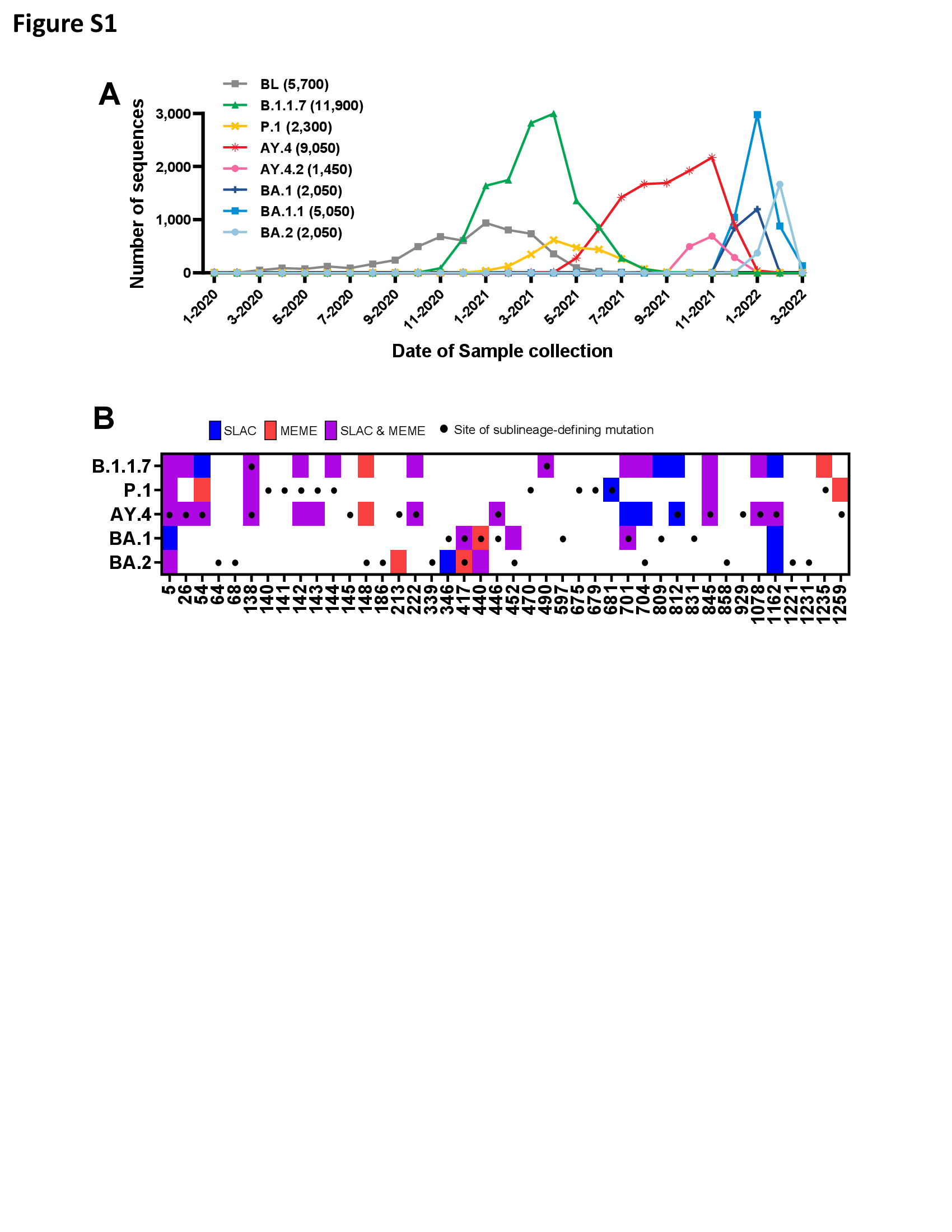

Supplement: S1 Fig — (A) Distribution of sample collection times for sequences of the indicated lineages and the SARS-CoV-2 baseline (BL) group. All sequences included in this study were unique, lacked any nucleotide ambiguities and appeared at least twice in the population (see numbers in parentheses). (B) Nucleotide sequences of isolates from the baselines of the indicated lineages were used to infer the rates of nonsynonymous and synonymous mutations by the Single-Likelihood Ancestor Counting (SLAC) method and by the Mixed Effects Model of Evolution (MEME) method. Sites assigned P-values smaller than 0.1 by either (or both) methods are colored as indicated. Black dots indicate sites of mutation that define VOC sublineages that emerged until April 8th 2022. (TIFF) [file pcbi.1012215.s001.tiff]
